# Supplementary material for: The actin modulator hMENA regulates GAS6‐AXL axis and pro‐tumor cancer/stromal cell cooperation
Source: EMBO Rep. 2020 Sep 10;21(11):e50078. doi: 10.15252/embr.202050078 (PMC7645265; doi:10.15252/embr.202050078)
Supplement: Supplementary file 1 — Appendix [file EMBR-21-e50078-s001.pdf]

Table of appendix PDF contents

1. Appendix Figures S1-S12 (pages 2-13)

2. Appendix Table S1 (page 14)

3. Appendix Table S2 (page 15)

4. Appendix Figure Legends (pages 16-20)

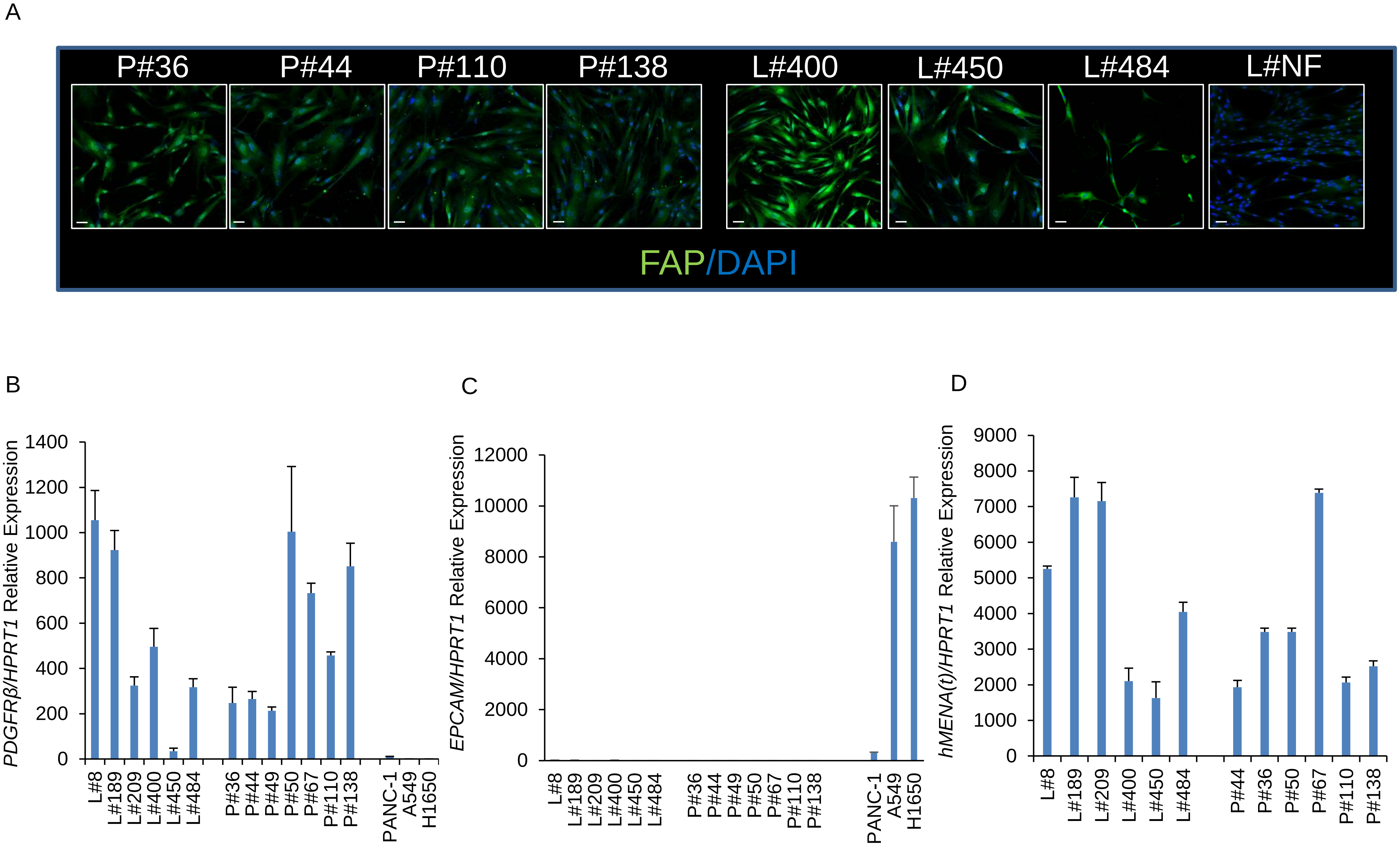

Appendix Figure S1

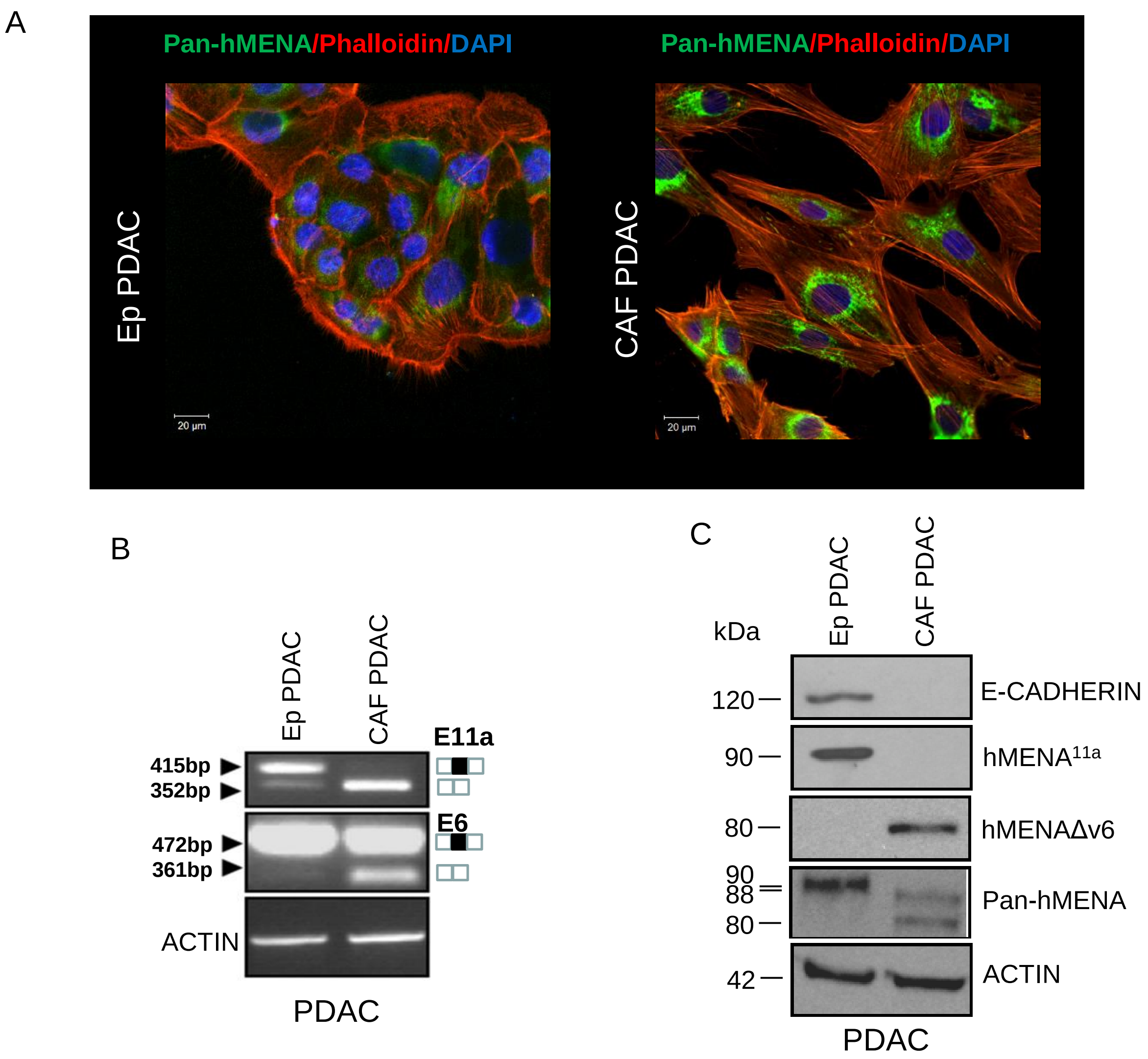

Appendix Figure S2

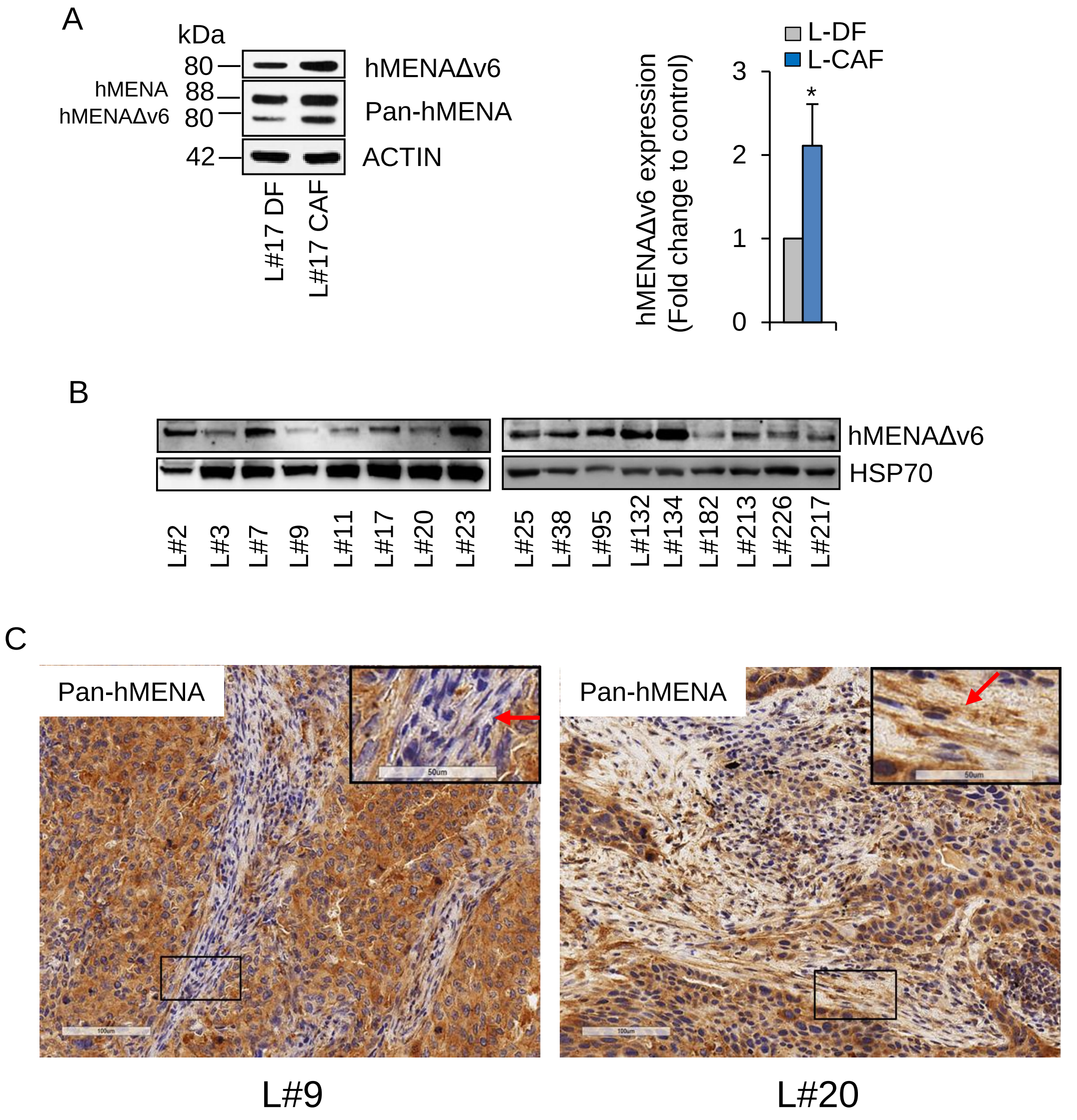

Appendix Figure S3

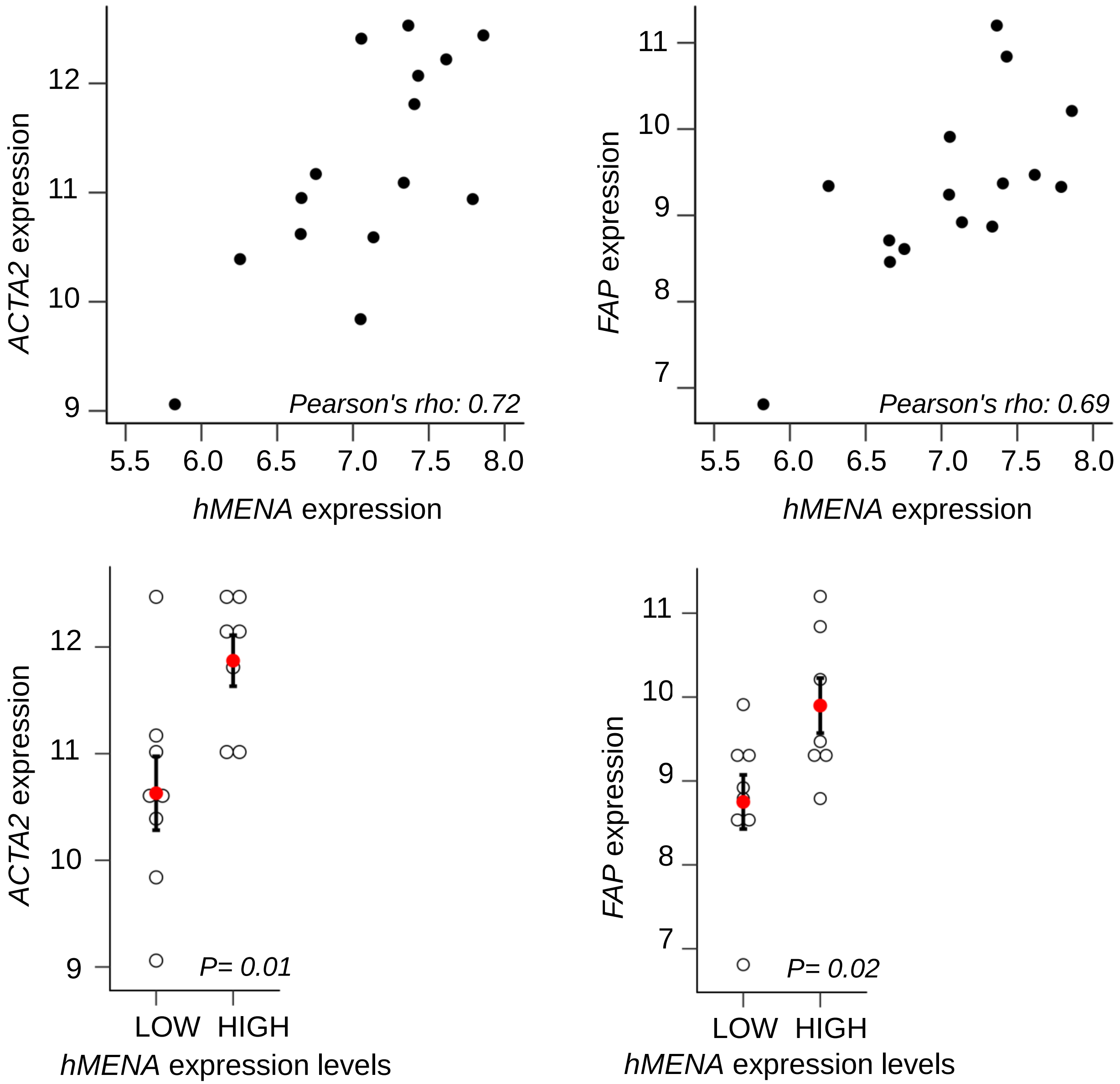

Appendix Figure S4

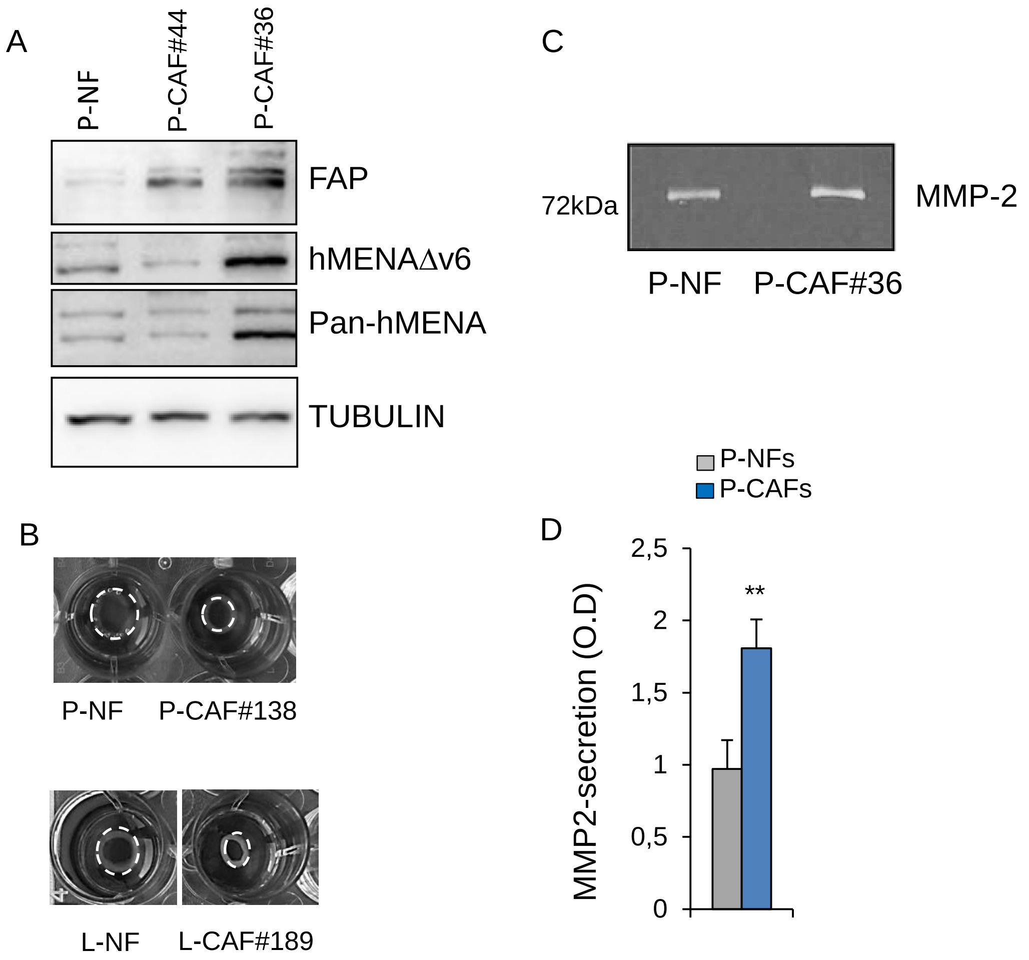

Appendix Figure S5

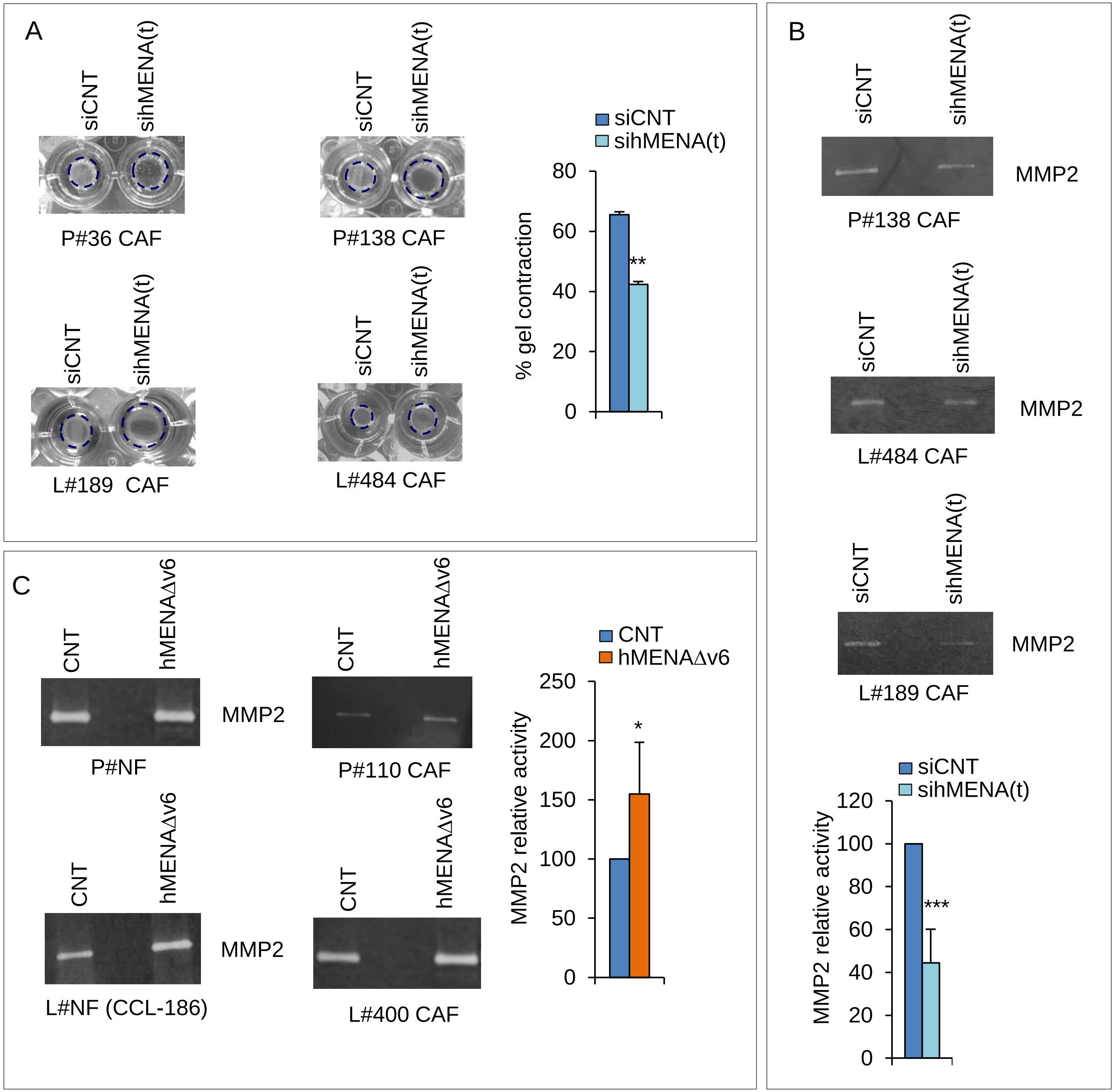

Appendix Figure S6

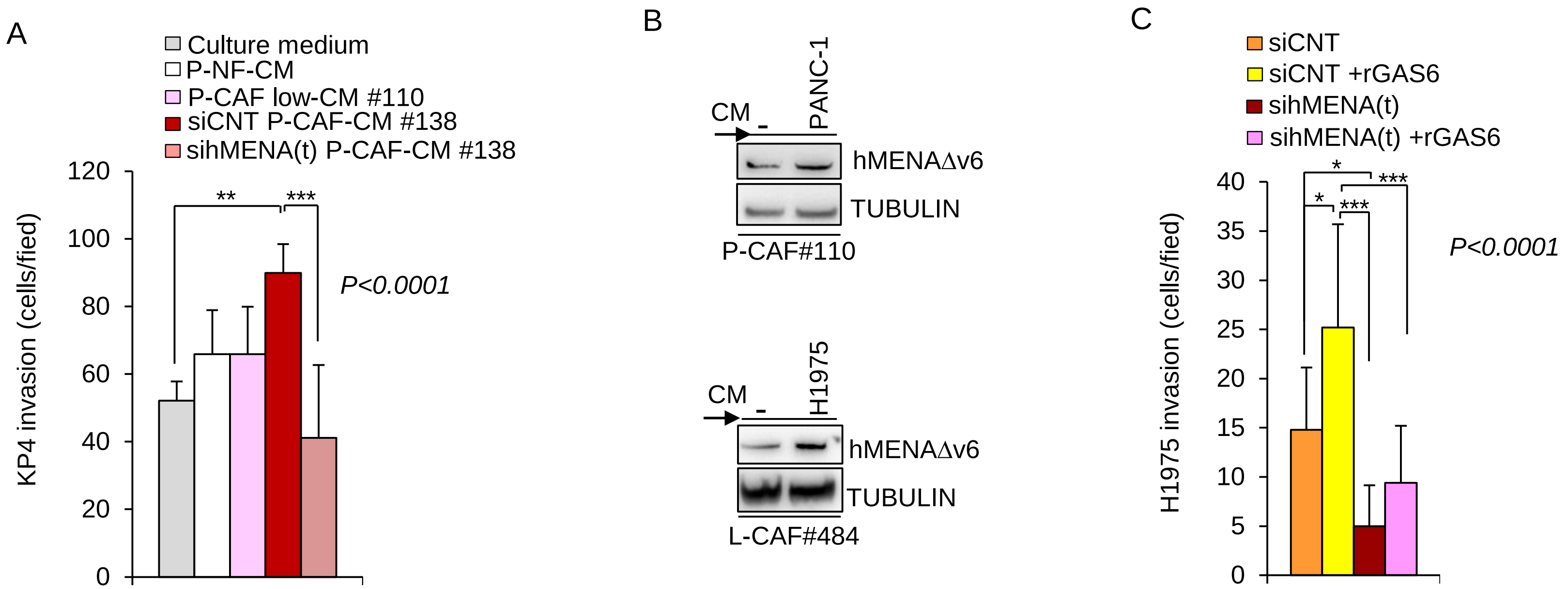

Appendix Figure S7

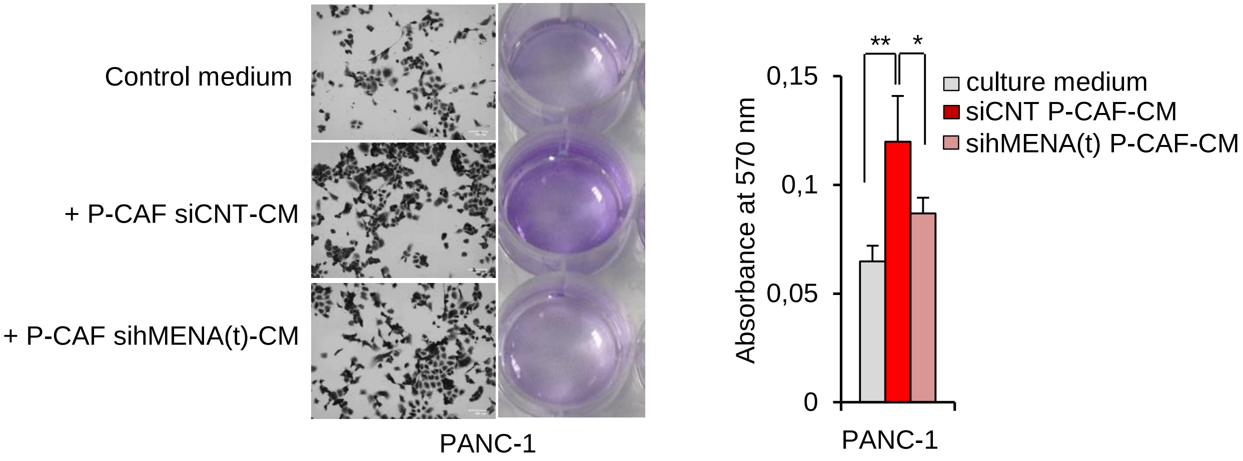

Appendix Figure S8

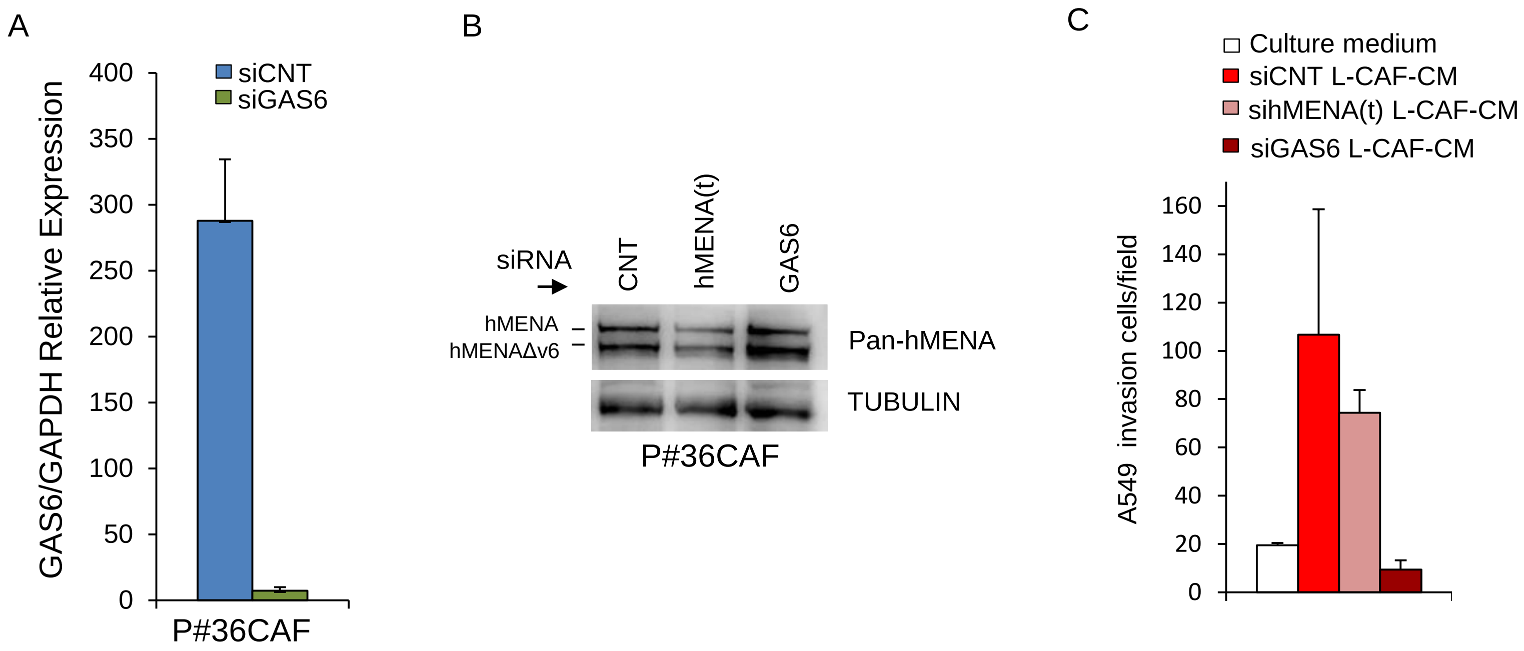

Appendix Figure S9

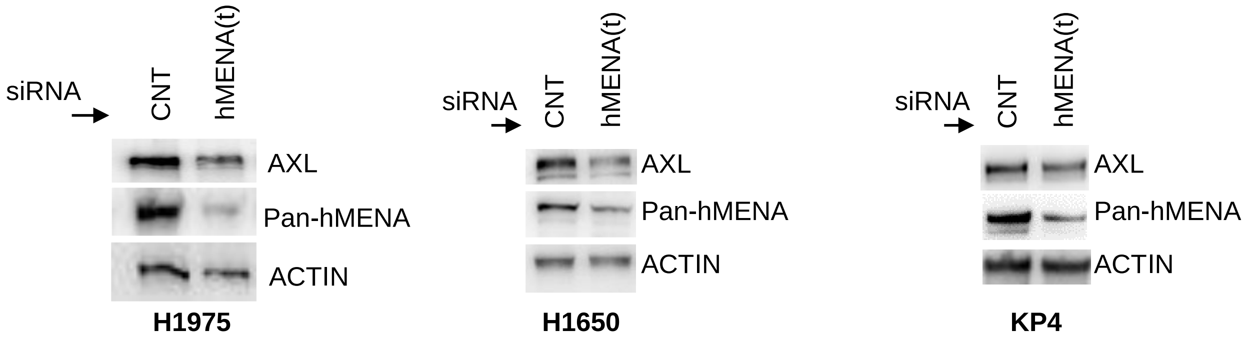

Appendix Figure S10

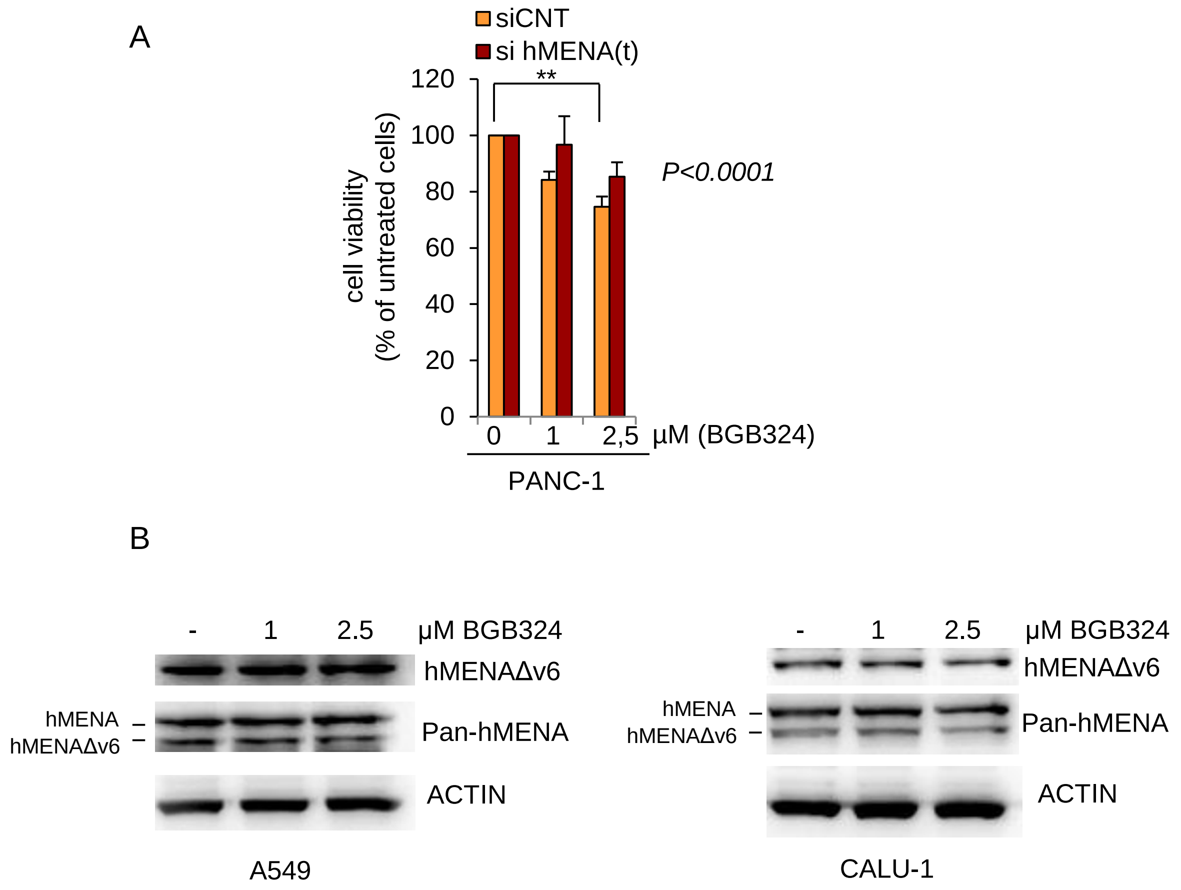

Appendix Figure S11

Pancreatic Adenocarcinoma patients

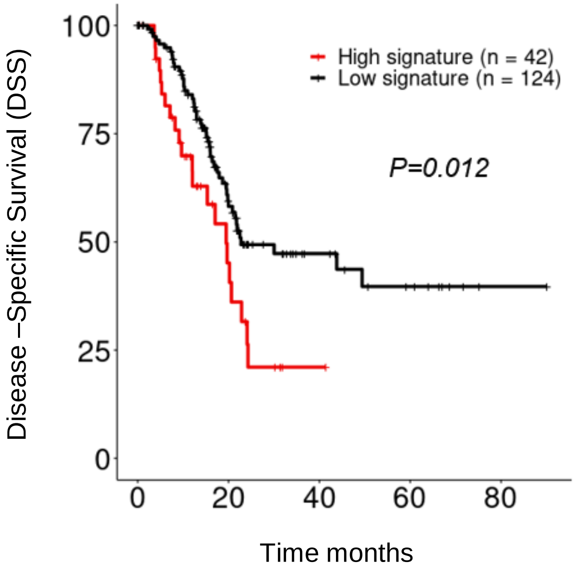

AXL-GAS6-ENAH genes signature

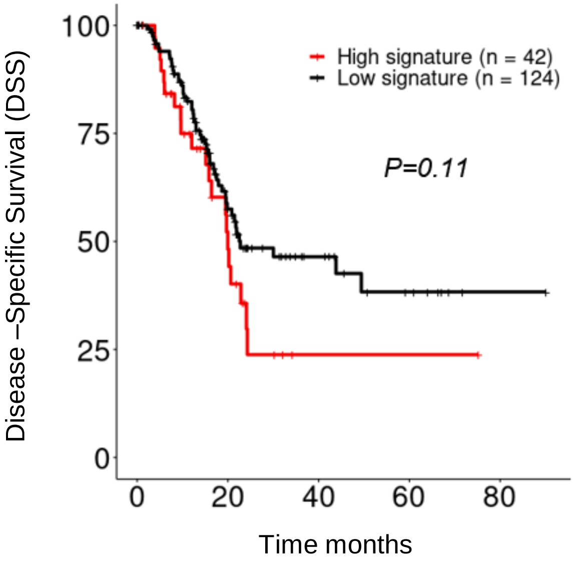

AXL-GAS6 genes signature

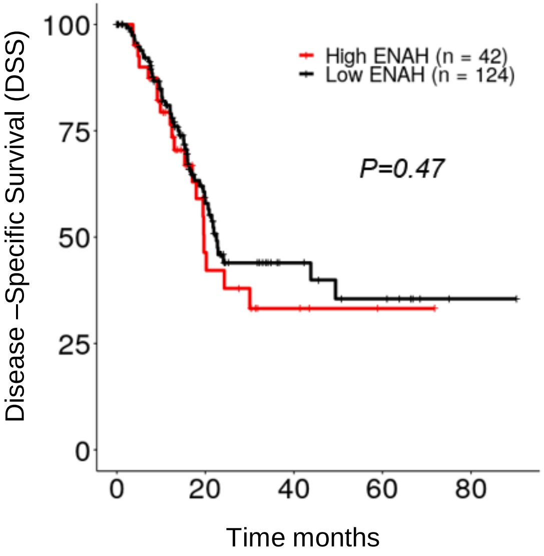

ENAH gene signature

Appendix Figure S12

Appendix Table S1. Clinical characteristics of *PDAC* tumors samples relative to P-CAFs primary cultures

Patients PDAC	Gender (F/M)	Age at diagnosis (years)	Histological Tumor type	Tumor Grade	TNM stage	Tumor size (mm)
**#036**	**M**	**63**	**ADC**	**G3**	**T3 N0**	**30**
**#044**	**M**	**59**	**ADC**	**G3**	**T3 N0**	**60**
#049	M	67	ADC	G2	T2 N0	30
**#050**	**M**	**57**	**ADC**	**G2-G3**	**T2 N1**	**40**
**#067**	**M**	**74**	**ADC**	**G2-G3**	**T2 N1**	**25**
#071	F	48	Serous Cystadenoma	-	-	30
#093	F	76	ADC	G3	nd	50
#097	F	58	Peritoneal Metastasis	nd	nd	nd
#106	M	76	ADC	G3	T3 N1	18
**#110**	**F**	**77**	**ADC**	**G2**	**T3 N0**	**15**
**#138**	**M**	**63**	**ADC**	**G3**	**T3N1**	**80**

Legend appendix Table S1. PDAC, Pancreatic Ductal Adenocarcinoma; ADC, Adenocarcinoma.

CAFs from tissues of patients #036; #044; #050; #067; #110; #138 (bolded) were evaluated by NGS with a panel of 22 genes (see Methods) and did not evidence any mutations.

Appendix Table S2. Clinical characteristics of *NSCLC* tumor samples relative to L-CAFs primary cultures

NSCLC Patients	Gender (F/M)	Age at diagnosis (years)	Histological Tumor type	Tumor Grade	TNM Stage	Tumor size (mm)	EGFR Mutations	ALK Mutations	K-RAS Mutations	RET Mutations	TP53 Mutations	B-RAF Mutations	CAF Mutations
#002	F	59	ADC	G2	T2a N0	42	wt	nd	wt	nd	nd	nd	nd
#003	M	77	ADC	G3	T2a N0	29	wt	nd	wt	nd	nd	nd	nd
#007	M	62	ADC	G3	T2a N0	45	wt	wt	wt	nd	nd	nd	nd
**#008**	**M**	**70**	**ADC**	**G2**	**T2bN0**	**30**	**wt**	**nd**	**p. G12V**	**nd**	**nd**	**nd**	**wt**
#009	M	57	ADC	G3	T2a N2	30	wt	wt	wt	nd	nd	nd	nd
#011	F	49	ADC	G3	T2b N2	70	wt	rearr 20%	wt	nd	nd	nd	nd
#017	M	65	ADC	G2-G3	T3 N2	52	wt	wt	wt	nd	nd	nd	nd
#020	M	78	SCC	G3	T1b N2	nd	nd	nd	nd	nd	nd	nd	nd
#023	M	71	ADC	G3	T1b N2	22	wt	wt	wt	nd	nd	nd	nd
#025	M	59	ADC	G3	T2a N0	50	wt	nd	wt	nd	nd	nd	nd
#038	M	69	ADC	G3	T2a N0	20	wt	nd	wt	nd	nd	nd	nd
#095	M	68	ADC	G3	T3 N2	100	wt	nd	p.G12D	nd	nd	nd	nd
#132	M	70	SCC	G3	T2a N2 Mx	40	nd	nd	nd	nd	nd	nd	nd
#134	M	71	ADC	G3	T2a N0	30	wt	nd	wt	nd	nd	nd	nd
#182	M	71	SCC	G2-G3	T3 N1	44	nd	nd	nd	nd	nd	nd	nd
#187	M	67	ADC	G2	T1b N0 V1 Pn0	21	wt	nd	p.G12A	nd	nd	nd	nd
**#189**	**M**	**70**	**ADC**	**G2**	**T1aN0**	**10**	**wt**	**nd**	**p. G12D**	**nd**	**nd**	**nd**	**wt**
**#209**	**M**	**60**	**ADC**	**G2**	**T1bN0**	**27**	**wt**	**nd**	**p. G12C**	**nd**	**nd**	**nd**	**wt**
#213	M	53	SCC	G3	T2a N0	40	nd	nd	nd	nd	nd	nd	nd
#217	M	58	SCC	G3	T3 N0	180	nd	nd	nd	nd	nd	nd	nd
#226	M	64	SCC	G3	T2b N0 V1 Pn0	52	nd	nd	nd	nd	nd	nd	nd
#227	F	78	ADC	G3	T2a N2	25	p.L858R	wt	wt	nd	nd	nd	nd
#310	M	71	SCC	G3	T3 N1	52	nd	nd	nd	nd	nd	nd	nd
**#400**	**F**	**48**	**ADC**	**G2**	**T1c N0**	**25**	**wt**	**wt**	**p. G12V**	**nd**	**nd**	**nd**	**wt**
**#450**	**M**	**58**	**ADC**	**G2**	**T2aN0**	**29**	**wt**	**wt**	**p. G12S**	**nd**	**nd**	**nd**	**wt**
**#484**	**M**	**48**	**ADC**	**G3**	**T2aN0**	**30**	**p. Leu747Ala750delinsPro**	**wt**	**wt**	**nd**	**p. Y220C**	**wt**	**wt**

Legend appendix Table S2. NSCLC, Non Small Cell Lung Cancer; ADC, Adenocarcinoma; SCC, Squamous Cell Carcinoma; wt, wilde type

CAFs from tissues of patients #008; #189; #209; #400; #450; #484 (bolded) were evaluated by NGS on a panel of 22 genes including genes mutated within cancer cells and did not evidence any of the mutations

Appendix Figure Legends

Appendix Figure S1

A. Representative images of IF analysis of P-CAFs, L-CAF and L-NFs stained with FAP Ab (green). Nuclei were stained with DAPI. Scale bar: 50 μm

B,C. Real time qRT-PCR analysis of *PDGFRβ* (B) and *EpCAM* (C) in P-CAFs and L-CAFs and in cancer cell lines (PANC-1, A549, H1650).

D. Real time qRT-PCR analysis of *ENAH* (hMENA) expression in in P-CAFs and L-CAFs.

Appendix Figure S2

A. Representative examples of IF analysis of EpPDAC and CAF PDAC cells, obtained from PDAC patient#36, stained with Pan-hMENA Ab (green) and phalloidin (red). The cell nuclei were stained with DAPI (blue).

B. qRT-PCR analysis of hMENA transcripts expression in EpPDAC and CAF-PDAC performed either with P7 forward and P8 reverse (Top) flanking the exon 11a or with primers MTC1 forward and MTC2 reverse flanking exon 6 (Middle). RT-PCR with primers specific for β-actin was performed as control of normalization (Bottom). PCR products were analyzed by agarose gel electrophoresis and ethidium bromide staining. Length of the amplified products are reported on the Left. E6, exon 6; E11a, exon 11a. exon 11a inclusion = □■□; 11a skipping = □□, or with exon 6 inclusion = □■□; exon 6 skipping = □□.

C. Representative immunoblot analysis of EpPDAC and CAF PDAC with E-cadherin, Pan-hMENA and hMENA isoform specific antibodies $hMENA^{11a}$ and hMENAΔv6. Actin was used as a loading control.

Appendix Figure S3

A. Representative immunoblot of hMENA/hMENAΔv6 expression levels in fibroblasts from “non tumoral” lung tissue obtained from patient #17, (L#17 DFs) and paired CAFs obtained from lung cancer tissue (L#17 CAFs) (left panel). Quantified data by densitometry represented as fold change of hMENAΔv6/ACTIN ratio in three different paired DF and CAF samples preparations (CAFs: #13,17,20) (right panel). Data are expressed as mean ± SD. P values were calculated by two-sided Student's t-test. *P< 0.05.

B. Immunoblot of hMENAΔv6 expression levels in L-CAFs (n=17). Patients from whom CAF were derived is indicated (#). HSP70 was used as loading control.

C. Representative IHC images of primary NSCLC tissues of patients #9 and #20 stained with Pan-hMENA mAb. Insets are higher magnification images of the indicated area showing that hMENA(t), is heterogeneously expressed in stromal cells with a morphology compatible with CAF as indicated by the arrows. Scale bar: 100 μm, inset: 50 μM.

Appendix Figure S4

Top. Shown are the correlations of hMENA (ENAH) mRNA expression with α-SMA (ACTA2) and FAP mRNA expression in primary NSCLC fibroblast cells (n=15). Pearson’s rho values are shown.

Bottom. Dotplots showing the mRNA expression of a-SMA (ACTA2) and FAP in primary NSCLC fibroblast cells (n=15). The samples were stratified into two groups on the basis of the hMENA mRNA levels, using as cut-off the median hMENA expression value as follows: low, below the median value; high, above the median value. Shown in each dotplot are the mean value (red dot), and standard deviations (vertical line). Statistical significance was calculated by two-tailed Student’s t-test. P values are shown.

Appendix Figure S5

A. Representative immunoblot analysis of FAP expression in P-NFs and P-CAFs. TUBULIN was used as a loading control.

B. Representative images of collagen gel contraction of P- and L-NF and P-#138 and L-#189 CAFs.

C. Representative image of gel zymography of MMP2 activity in P-NF and P-CAFs #36. Similar results were obtained in two independent experiments.

D. Quantification by ELISA of MMP-2 secreted level in the CM of pancreatic normal fibroblasts P-NF), and PDAC CAFs (P-CAF). Representative results from 2 independent experiments are shown. Data are expressed as mean ± SD. P value was calculated by two-sided Student's t-test. **P< 0.01.

Appendix Figure S6

A. Representative images and quantification of collagen gel contraction ability in sihMENA(t) P-CAFs (#36,138) and L-CAFs (#189,484) with respect to siCNT CAFs, indicating that the siRNA-mediated knock-down of hMENA/hMENAΔv6 reduces the collagen gel contraction ability of CAFs with respect to siCNT CAFs. Quantification data represent the mean ± SD of the 4 CAF samples (#36,#138,#189,#484) siCNT and sihMENA(t). P value was calculated by two-sided Student's t-test. **P< 0.01.

B. Representative images and quantification of gel zymography showing decreased MMP2 activity in sihMENA(t) P- CAFs (#138) and L-CAFs (#189,484) with respect to siCNT CAFs (set as 100).

Quantification data represent the mean ± SD of the 3 CAF samples (P#138, L#484, L#189,) siCNT and si hMENA(t). P value was calculated by two-sided Student's t-test. ***P< 0.001.

C. Representative images and quantification of gel zymography of P-and L-NFs, P-CAFs (#110) and L-CAFs (#400) transfected with control or hMENAΔ v6 expressing vectors, showing an increased MMP2 activity in fibroblasts overexpressing hMENAΔv6 isoform compared to control cells (CNT, set as 100). Quantification data represent the mean ± SD of the 4 CAF samples (P-and L-NFs, P-CAFs (#110) and L-CAFs (#400), CNT and hMENAΔv6. P value was calculated by two-sided Student's t-test. *P< 0.05.

Appendix Figure S7

A. Quantification of in vitro matrigel invasion assay of KP4 PDAC cells cultured for 48 h with DMEM (culture medium), conditioned media (CM) of: NFs (P-NF-CM); CAF low P#110 CAF; CAF high P#138 control (siCNT) or silenced for hMENA(t). Histograms show the number of invading cells measured by counting 6 random fields. Statistical analysis was performed with one-way ANOVA P=0.004, followed by Bonferroni's multiple comparison test. **P<0.01, *** P< 0.001.

B. Representative immunoblot of hMENAΔv6 expression level in P-CAF#110 (up) and L-CAF#484 (bottom) cultured for 24 h in the presence of control medium (-) or PANC-1–CM and H1975-CM respectively.

C. Quantification of in vitro matrigel invasion assay of H1975 siCNT cells (siCNT) and hMENA/hMENAΔv6 silenced cells sihMENA(t) toward rGAS6 as chemo-attractant, showing that the knock-down of hMENA(t) reduced cancer cell invasion toward GAS6. The number of invading cells were counted in 6 random fields. Statistical analysis was performed with one-way ANOVA P=0.004, followed by Bonferroni's multiple comparison test. *P<0.05, *** P< 0.001.

Appendix Figure S8

Representative images (left) and quantification (right) of PANC-1 cells growth for 24 h in the presence of culture medium or conditioned media derived from P#106 CAF transfected with control siRNA (+ P-CAF siCNT-CM) or hMENA(t)siRNA (P-CAF sihMENA(t)-CM). PANC-1 growth was examined by crystal violet staining. P value was calculated by two-sided Student's t-test. *P< 0.05, **P<0.01.

Appendix Figure S9

A. Real time qRT-PCR analysis of relative GAS6 mRNA expression level in P-CAF transfected with control siRNA (siCNT) or GAS6 siRNA (siGAS6).

B. Representative immunoblot analysis of hMENAΔv6 expression and total hMENA isoforms (pan-hMENA) in P-CAF transfected with control siRNA (CNT), hMENA(t) siRNA, hMENA(t), or GAS6 siRNA (GAS6).

C. Quantification of matrigel invasion assay of A549 cells cultured for 48 h in the presence of cultue medium (culture medium), CM derived from CAFs transfected with control siRNA (siCNT L-CAF-CM), hMENA(t) siRNA (sihMENA(t) L-CAF-CM), GAS6 siRNA (siGAS6 L-CAF-CM). Number of invaded cells was measured by counting 6 random fields.

Appendix Figure S10

Representative immunoblot analysis, of two independent experiments, of AXL and total hMENA expression (Pan-hMENA) in NSCLC H1975, H1650 and PDAC KP4 cell lines upon transfection with control siRNA (CNT) and hMENA(t) siRNA, sihMENA(t).

Appendix Figure S11

A. Quantification, by crystal violet staining, of cell viability of PANC-1 cell transfected with control siRNA (siCNT) or hMENA(t) siRNA, sihMENA(t) and treated for 24 h with vehicle (DMSO) or BGB324 at 1, 2,5 μM concentrations.

Data are presented as percentage of crystal violet staining compared to untreated control (set as 100) and are mean ± SD (n=3). Statistical analysis was performed with one-way ANOVA P<0.0001, followed by Bonferroni's multiple comparison test. **P<0.01.

B. Immunoblot analysis of hMENAΔv6 and total hMENA isoform expression (Pan-hMENA) and in NSCLC cancer cell lines A549 and CALU-1, treated with DMSO (-) or BGB324 at two different concentrations (1 and 2,5 uM) for 48 h.

Appendix Figure S12

Disease Specific Survival (DSS) curves in Pancreatic Adenocarcinoma patients (PDAC) (n=172) from The Cancer Genome Atlas (TCGA).

Patients were stratified in two groups on the basis of the AXL/GAS6/ENAH (left), AXL/GAS6 (middle) and ENAH (right) signature expression levels. P values are shown. Statistical significance was calculated by using the log-rank test.
