# Supplementary material for: The actin modulator hMENA regulates GAS6‐AXL axis and pro‐tumor cancer/stromal cell cooperation
Source: EMBO Rep. 2020 Sep 10;21(11):e50078. doi: 10.15252/embr.202050078 (PMC7645265; doi:10.15252/embr.202050078)
Supplement: Supplementary file 2 — Source Data for Figure 1 [file EMBR-21-e50078-s002.pptx]

## Slide 1
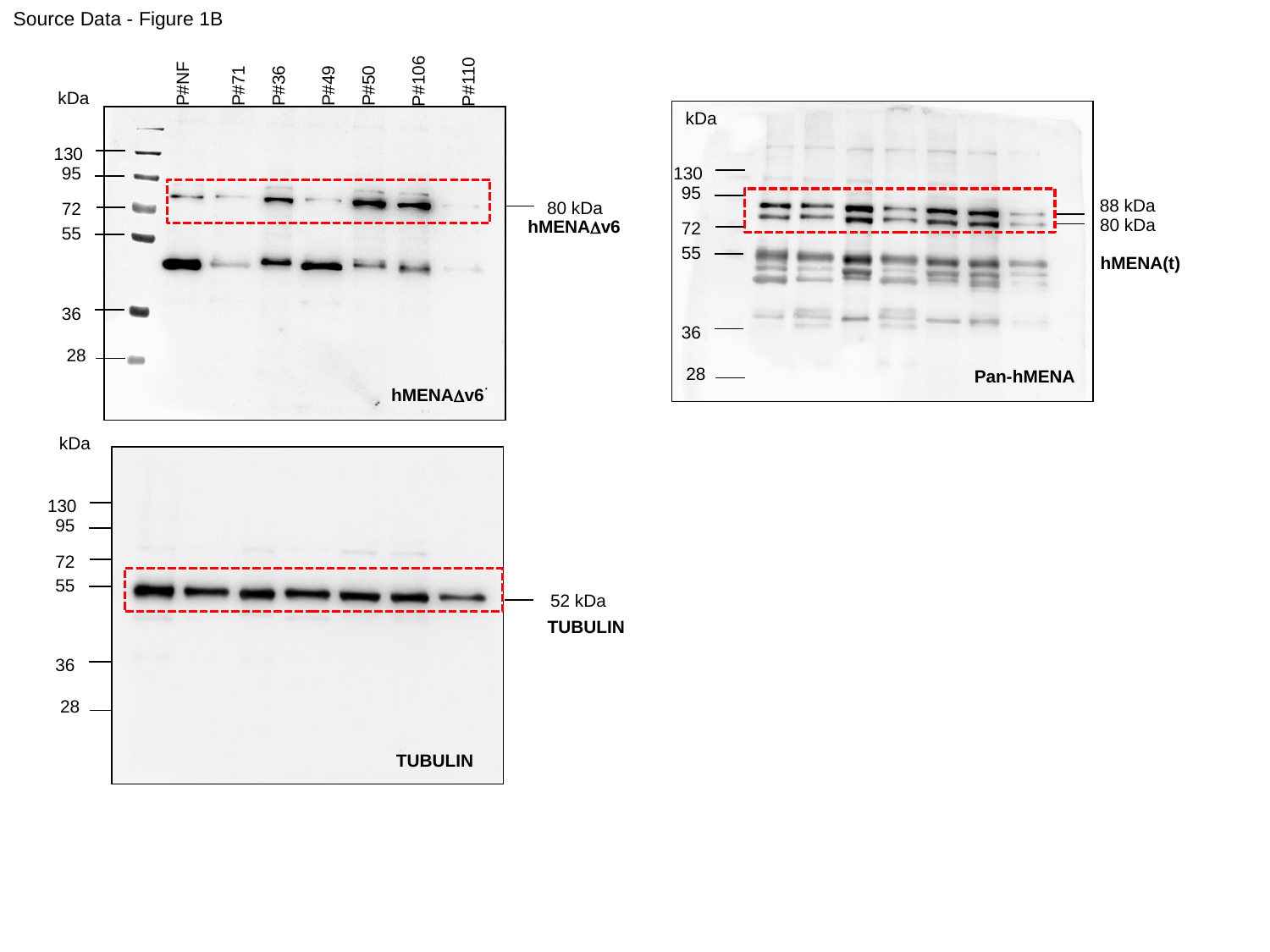

Source Data - Figure 1B
P#110
P#106
P#NF
P#71
P#36
P#49
P#50
kDa
kDa
130
95
72
55
36
28
130
95
72
55
36
28
88 kDa
80 kDa
hMENA(t)
80 kDa
hMENAΔv6
Pan-hMENA
hMENAΔv6
kDa
130
95
72
55
36
28
52 kDa
TUBULIN
TUBULIN

## Slide 2
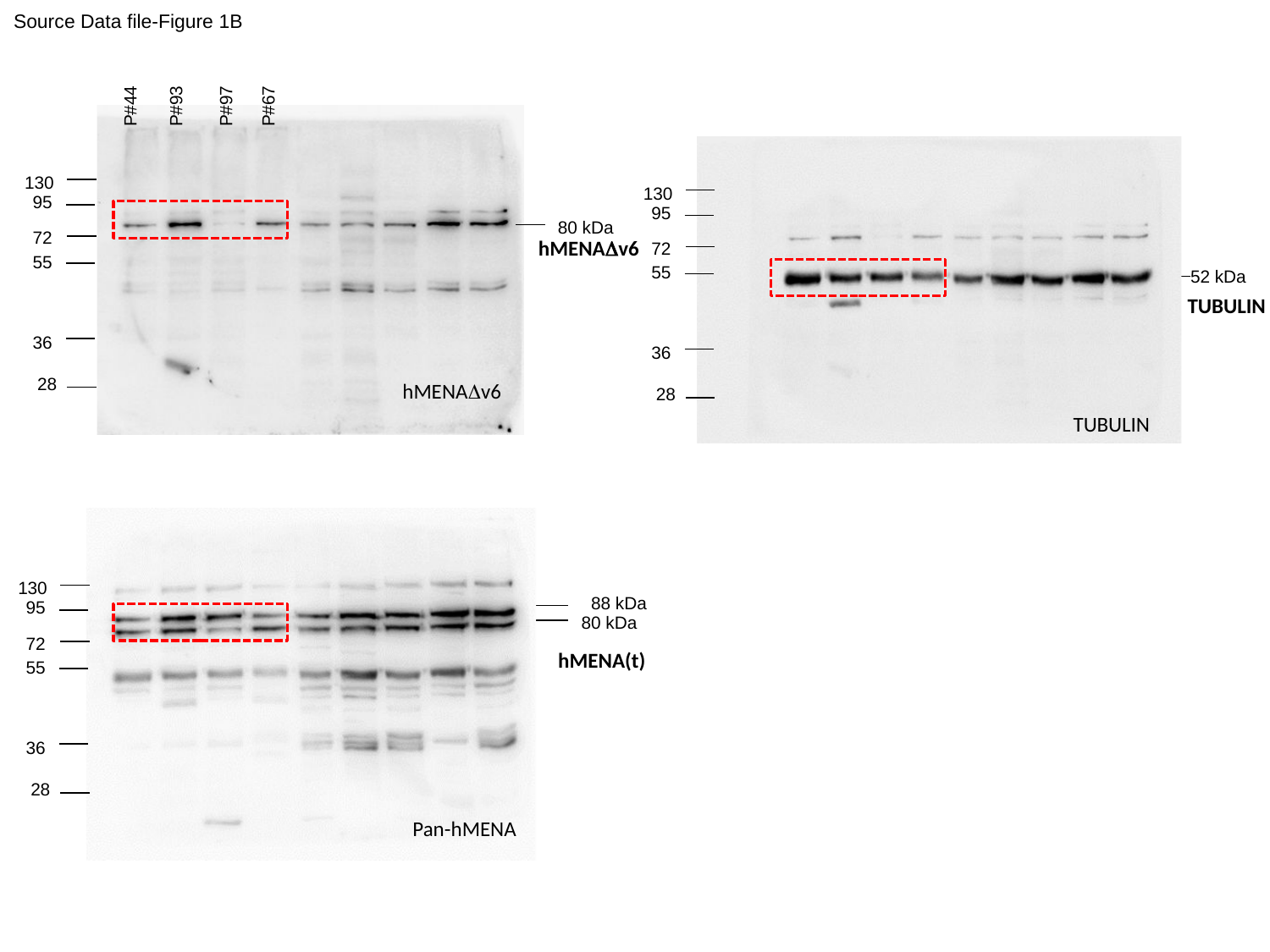

Source Data file-Figure 1B
P#44
P#93
P#97
P#67
130
95
72
55
36
28
130
95
72
55
36
28
80 kDa
hMENAΔv6
52 kDa
TUBULIN
hMENAΔv6
TUBULIN
130
95
72
55
36
28
88 kDa
80 kDa
hMENA(t)
Pan-hMENA

## Slide 3
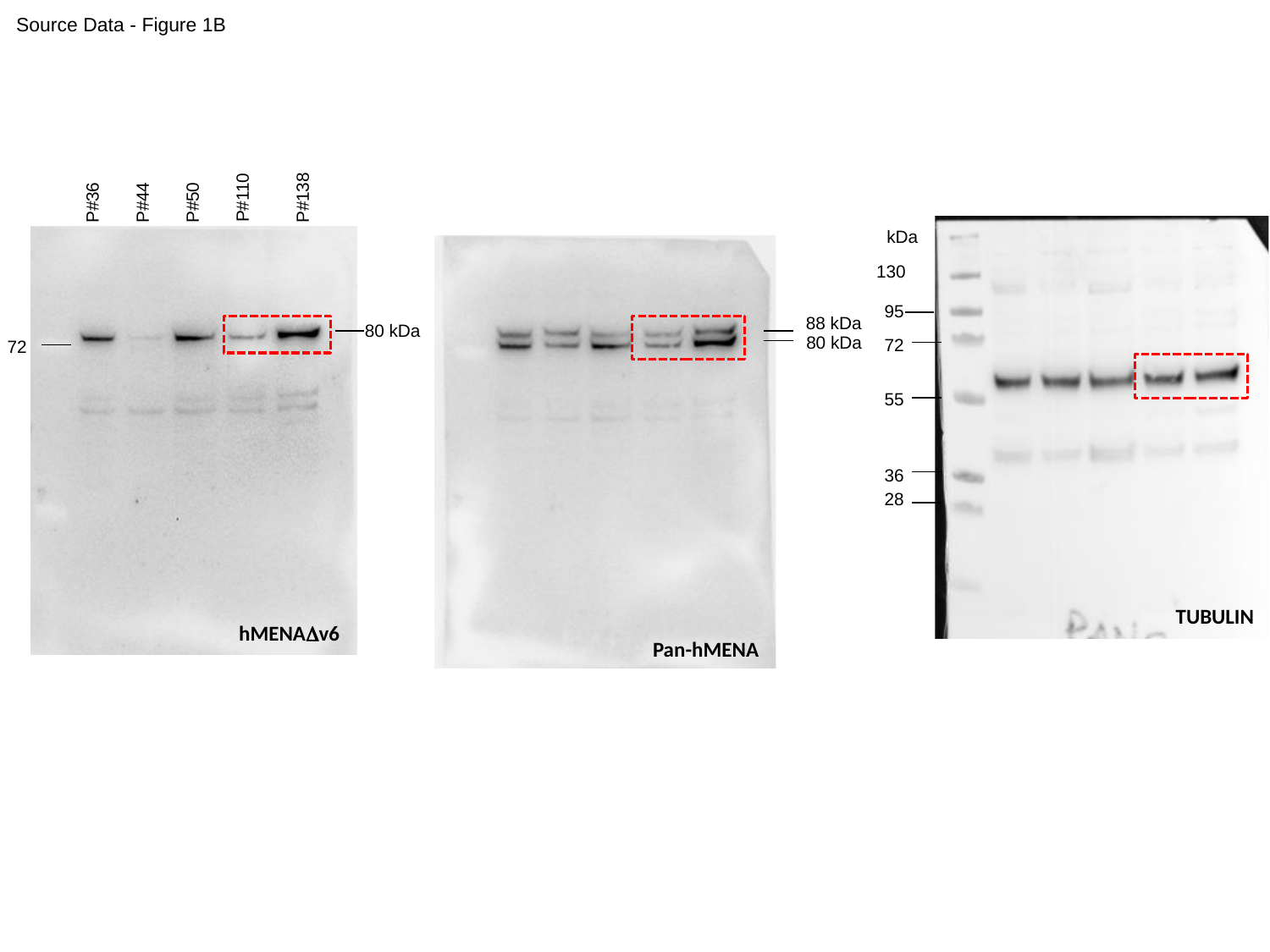

Source Data - Figure 1B
P#110
P#138
P#36
P#44
P#50
kDa
88 kDa
80 kDa
130
95
80 kDa
72
72
55
36
28
TUBULIN
hMENAΔv6
Pan-hMENA

## Slide 4
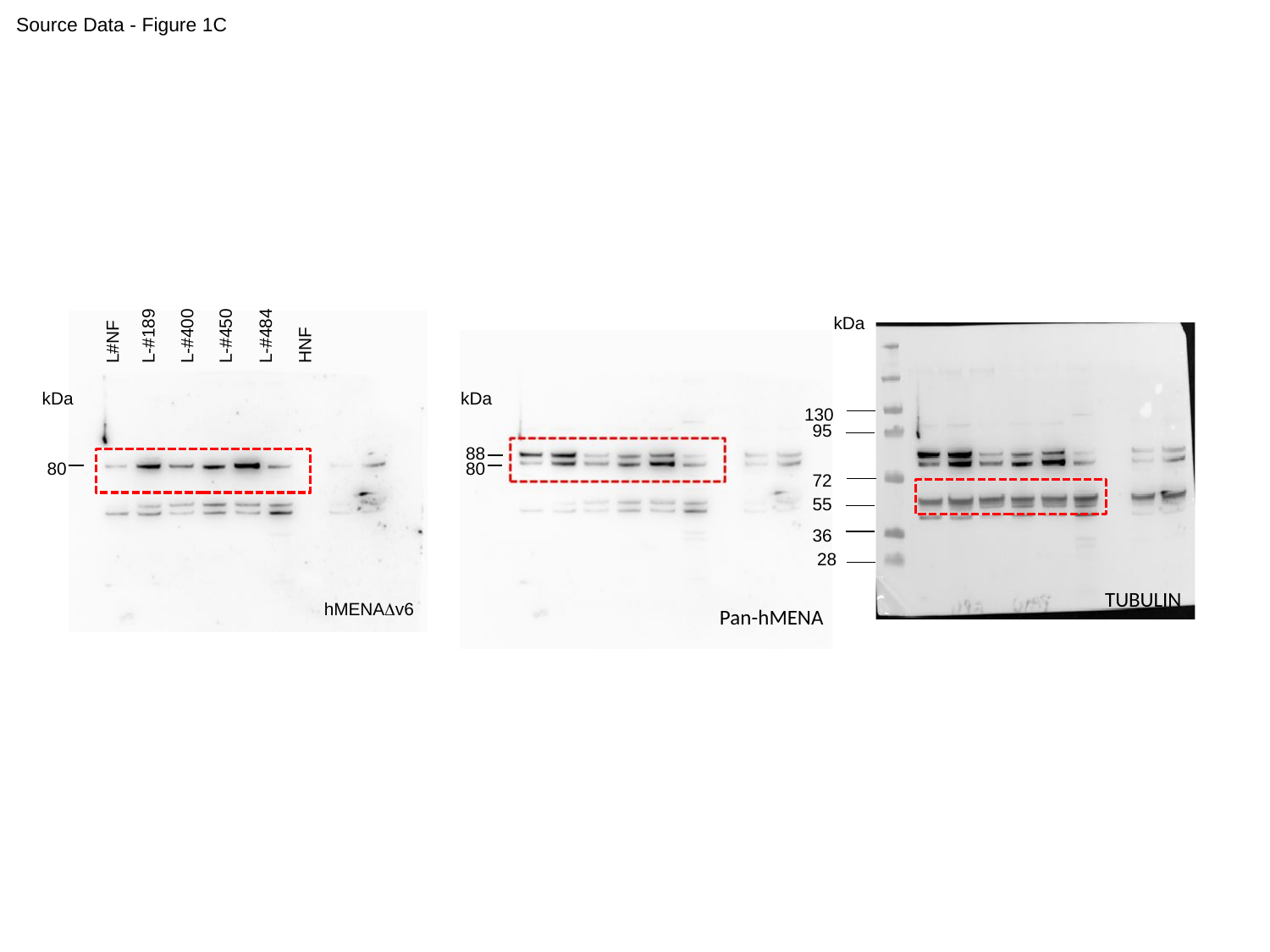

Source Data - Figure 1C
kDa
hMENAΔv6
kDa
80
L-#189
L-#400
L-#450
L-#484
TUBULIN
TUBULIN
L#NF
HNF
L-#189
L-#400
L-#450
L-#484
kDa
88
80
L#NF
HNF
130
95
72
55
36
28
Pan-hMENA
