# Supplementary material for: The actin modulator hMENA regulates GAS6‐AXL axis and pro‐tumor cancer/stromal cell cooperation
Source: EMBO Rep. 2020 Sep 10;21(11):e50078. doi: 10.15252/embr.202050078 (PMC7645265; doi:10.15252/embr.202050078)
Supplement: Supplementary file 3 — Source Data for Figure 3 [file EMBR-21-e50078-s003.pptx]

## Slide 1
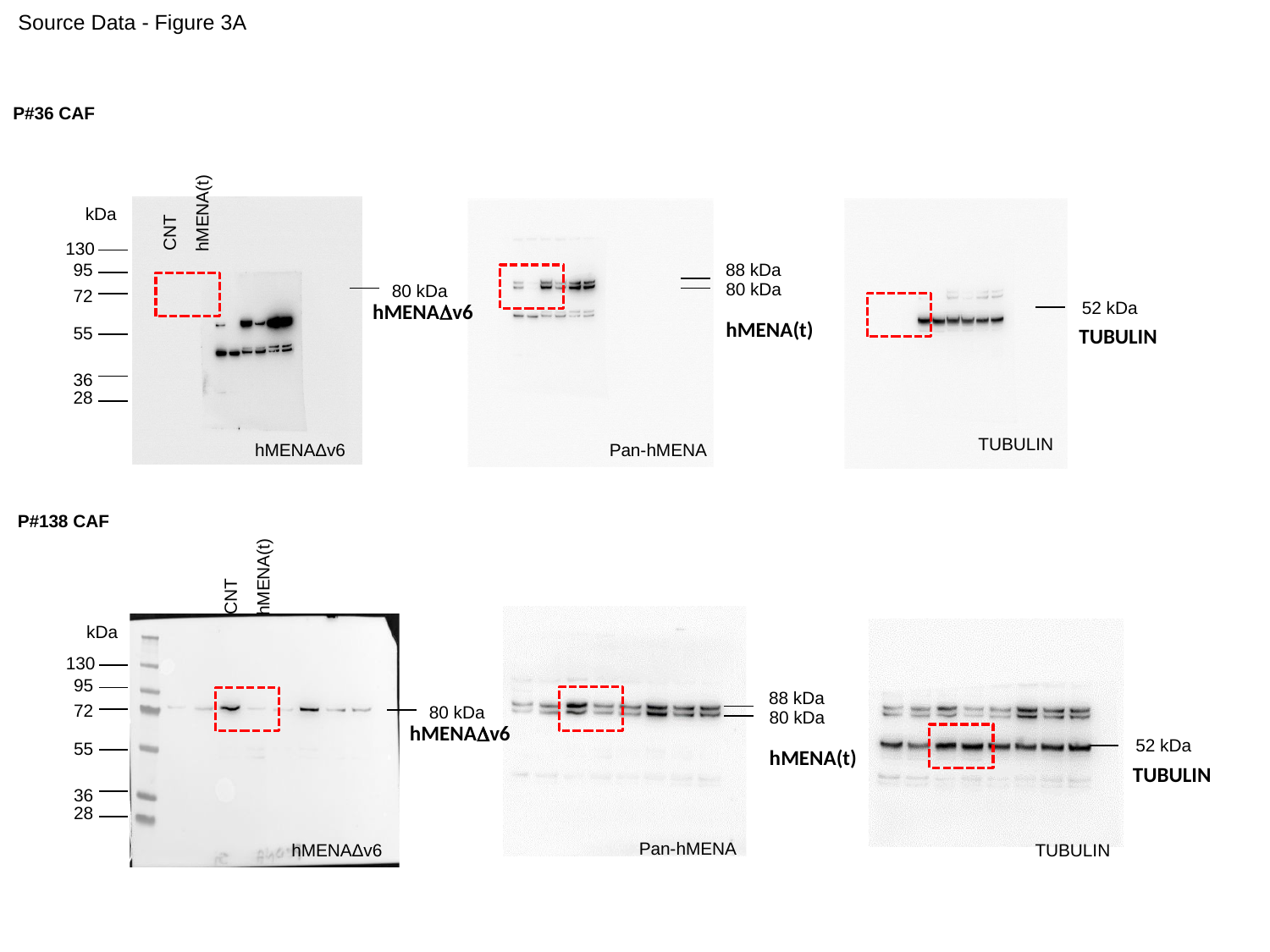

Source Data - Figure 3A
P#36 CAF
hMENA(t)
kDa
CNT
130
95
72
55
36
28
88 kDa
80 kDa
hMENA(t)
80 kDa
hMENAΔv6
52 kDa
TUBULIN
TUBULIN
hMENAΔv6
Pan-hMENA
P#138 CAF
hMENA(t)
CNT
kDa
130
95
72
55
36
28
88 kDa
80 kDa
hMENA(t)
80 kDa
hMENAΔv6
52 kDa
TUBULIN
Pan-hMENA
hMENAΔv6
TUBULIN

## Slide 2
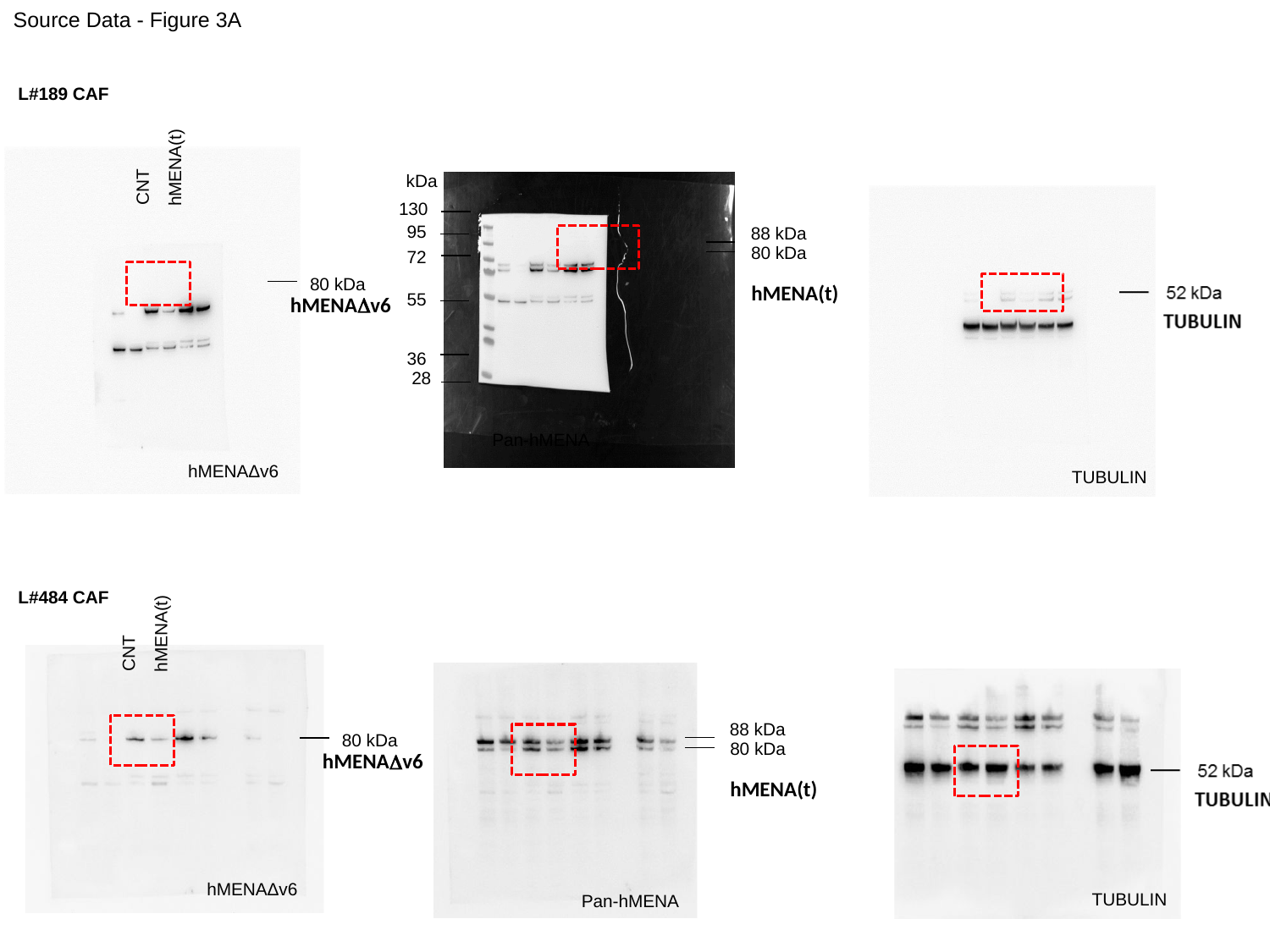

Source Data - Figure 3A
L#189 CAF
hMENA(t)
kDa
CNT
TUBULIN
130
95
88 kDa
80 kDa
hMENA(t)
72
80 kDa
hMENAΔv6
55
36
28
Pan-hMENA
hMENAΔv6
L#484 CAF
hMENA(t)
CNT
88 kDa
80 kDa
hMENA(t)
80 kDa
hMENAΔv6
hMENAΔv6
TUBULIN
Pan-hMENA

## Slide 3
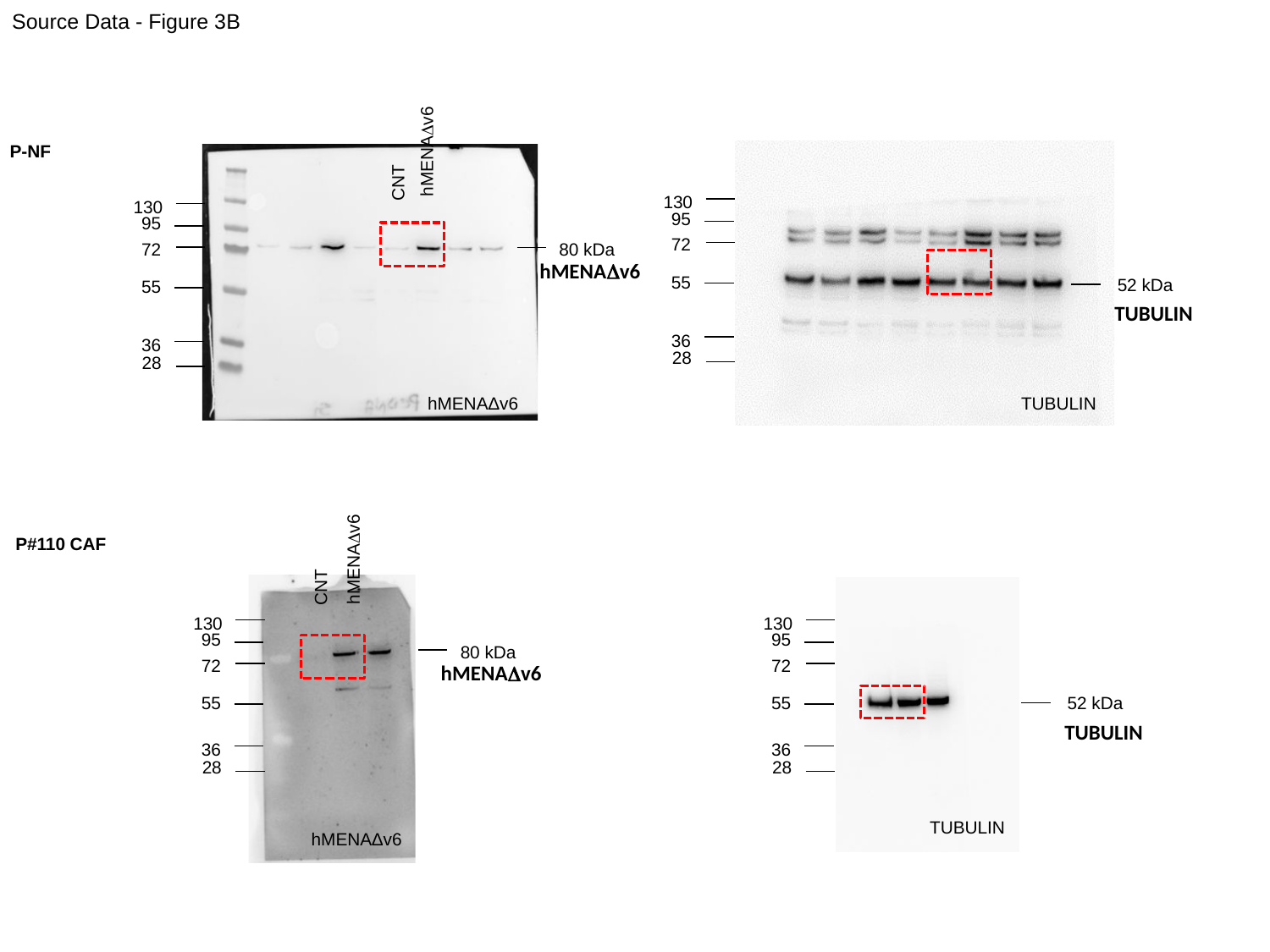

Source Data - Figure 3B
P-NF
hMENAΔv6
CNT
130
95
72
55
36
28
130
95
72
55
36
28
80 kDa
hMENAΔv6
52 kDa
TUBULIN
TUBULIN
hMENAΔv6
P#110 CAF
hMENAΔv6
CNT
130
95
72
55
36
28
130
95
72
55
36
28
80 kDa
hMENAΔv6
52 kDa
TUBULIN
TUBULIN
hMENAΔv6

## Slide 4
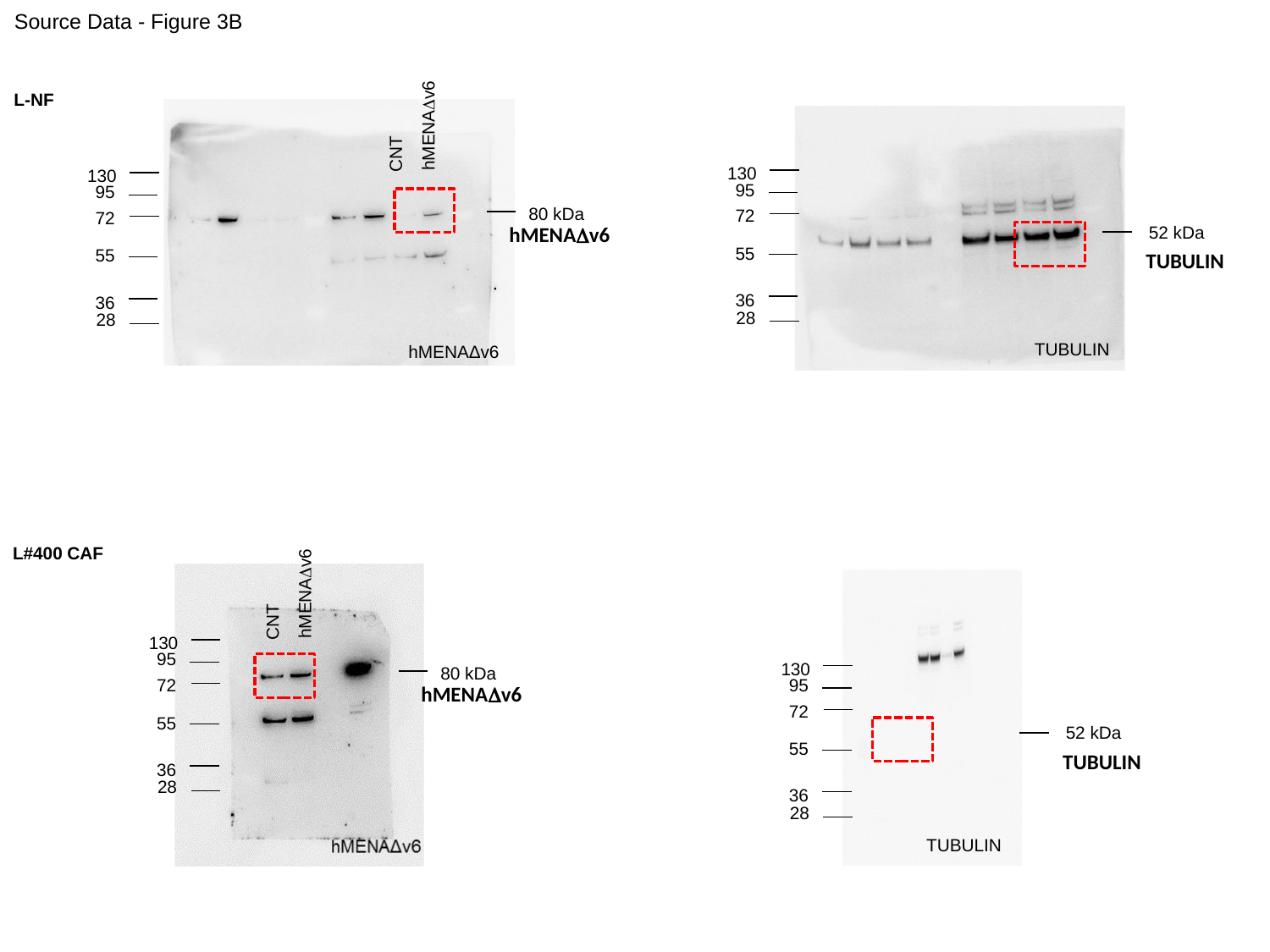

Source Data - Figure 3B
L-NF
hMENAΔv6
CNT
130
95
72
55
36
28
130
95
72
55
36
28
80 kDa
hMENAΔv6
52 kDa
TUBULIN
TUBULIN
hMENAΔv6
L#400 CAF
hMENAΔv6
CNT
130
95
72
55
36
28
130
95
72
55
36
28
80 kDa
hMENAΔv6
52 kDa
TUBULIN
TUBULIN
