# Supplementary material for: The actin modulator hMENA regulates GAS6‐AXL axis and pro‐tumor cancer/stromal cell cooperation
Source: EMBO Rep. 2020 Sep 10;21(11):e50078. doi: 10.15252/embr.202050078 (PMC7645265; doi:10.15252/embr.202050078)
Supplement: Supplementary file 4 — Source Data for Figure 4 [file EMBR-21-e50078-s004.pptx]

## Slide 1
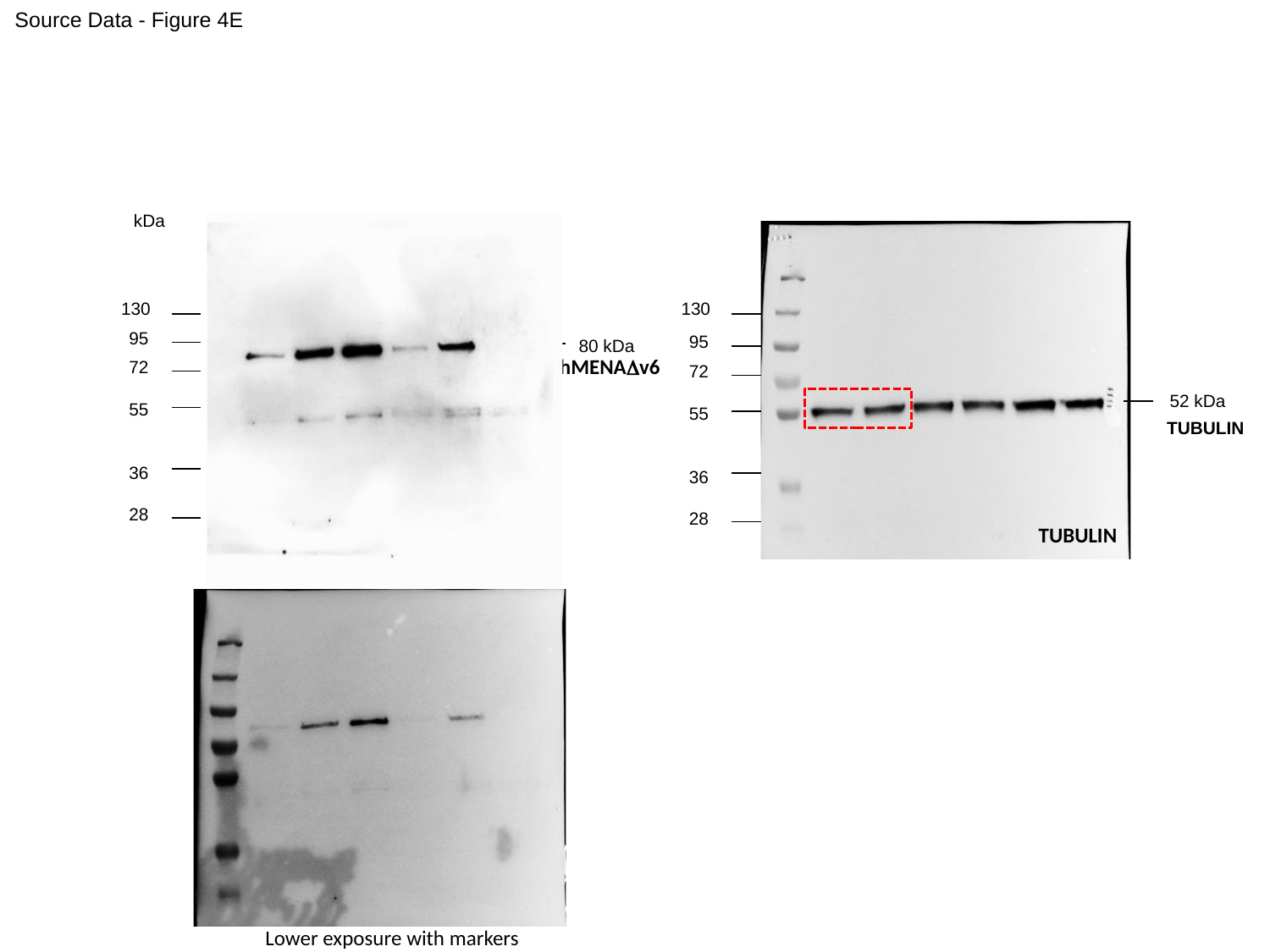

Source Data - Figure 4E
kDa
130
130
95
95
80 kDa
hMENAΔv6
72
72
52 kDa
55
55
TUBULIN
36
36
28
28
hMENAΔv6
TUBULIN
Lower exposure with markers

## Slide 2
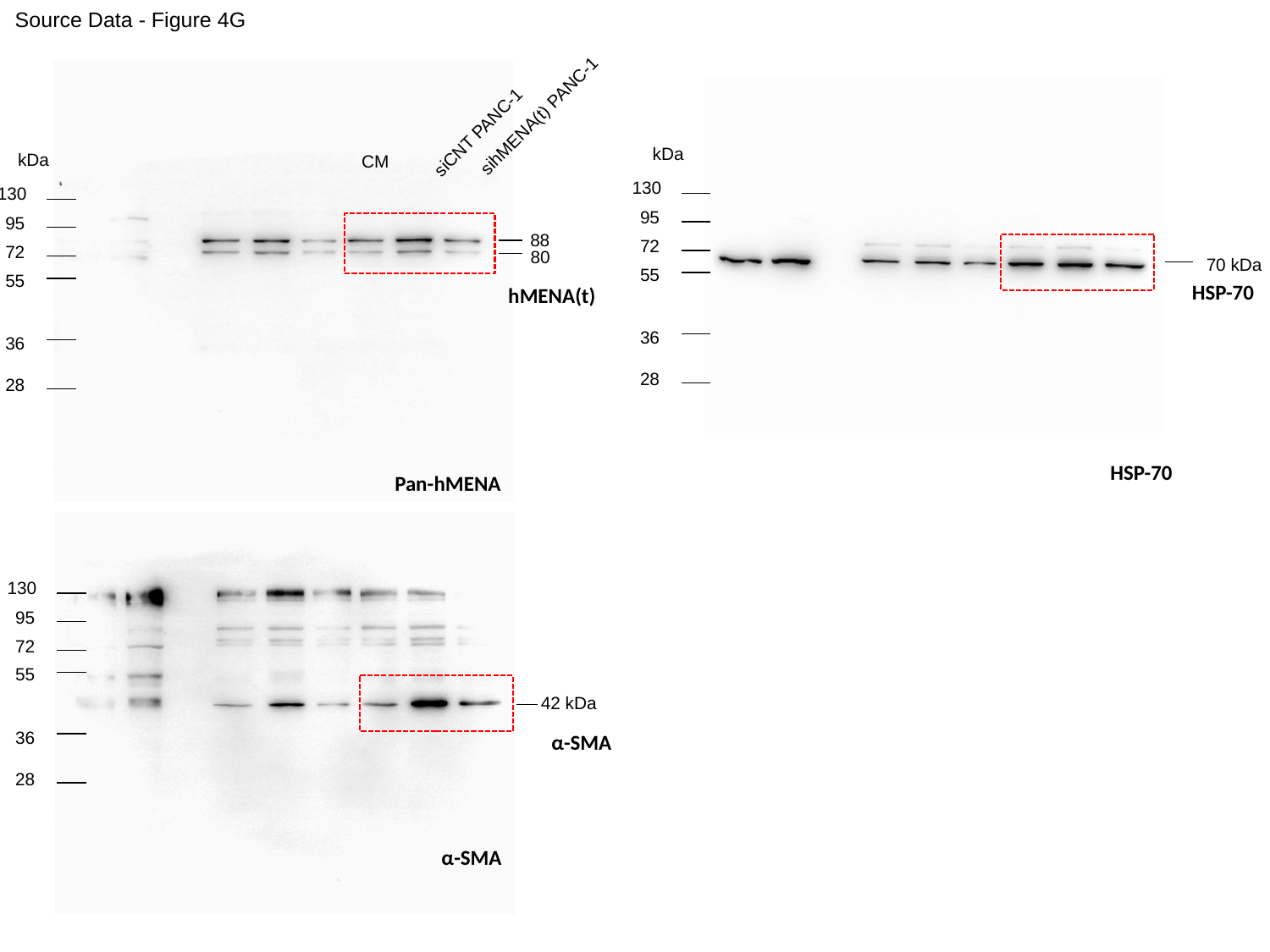

Source Data - Figure 4G
sihMENA(t) PANC-1
siCNT PANC-1
kDa
kDa
CM
130
95
72
55
36
28
130
95
72
55
36
28
88
80
hMENA(t)
70 kDa
HSP-70
HSP-70
Pan-hMENA
130
95
72
55
36
28
42 kDa
α-SMA
α-SMA
