# Supplementary material for: The actin modulator hMENA regulates GAS6‐AXL axis and pro‐tumor cancer/stromal cell cooperation
Source: EMBO Rep. 2020 Sep 10;21(11):e50078. doi: 10.15252/embr.202050078 (PMC7645265; doi:10.15252/embr.202050078)
Supplement: Supplementary file 5 — Source Data for Figure 6 [file EMBR-21-e50078-s005.pptx]

## Slide 1
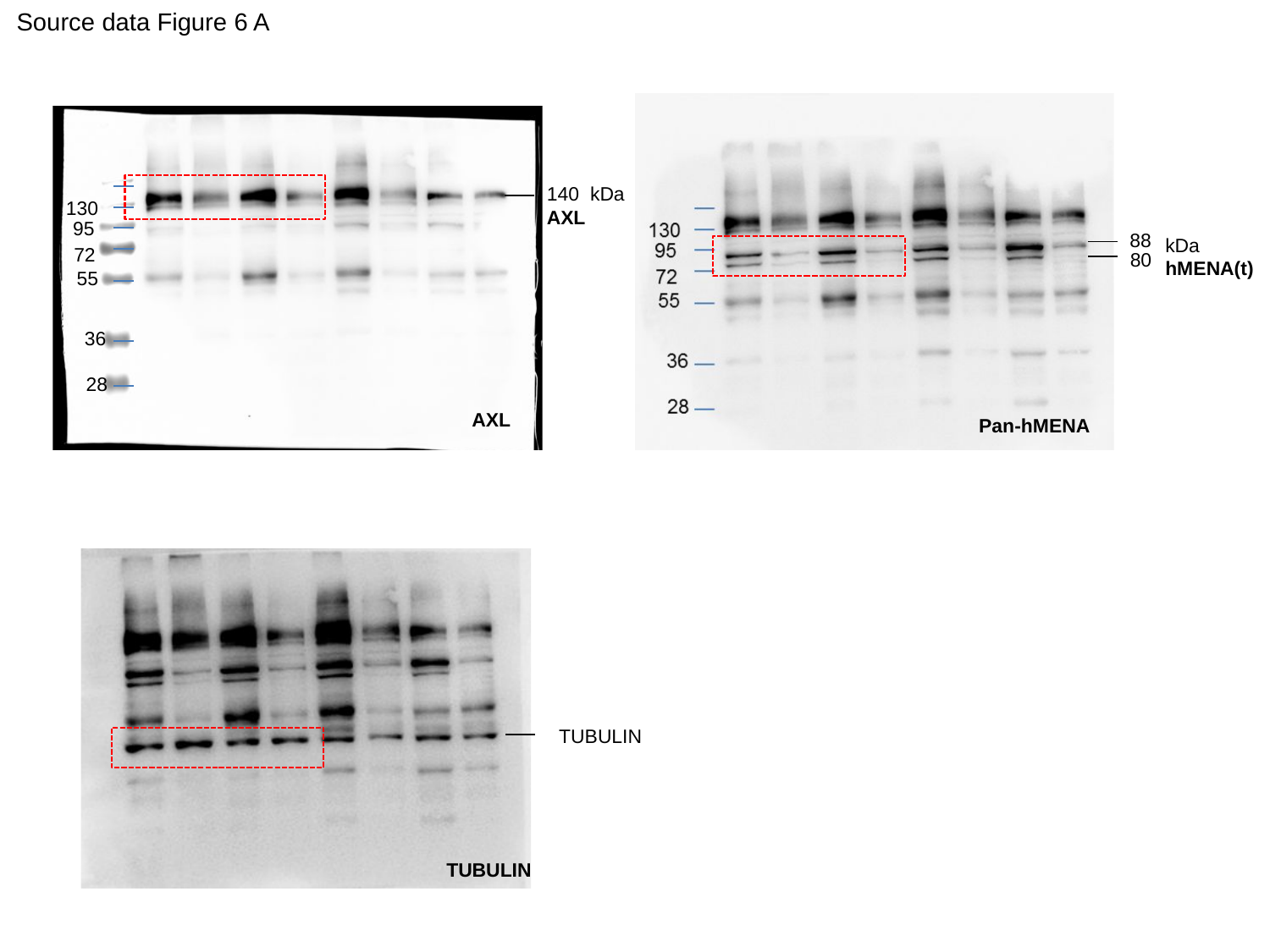

Source data Figure 6 A
140 kDa
AXL
130
95
72
55
36
28
88
kDa
hMENA(t)
80
AXL
Pan-hMENA
TUBULIN
TUBULIN

## Slide 2
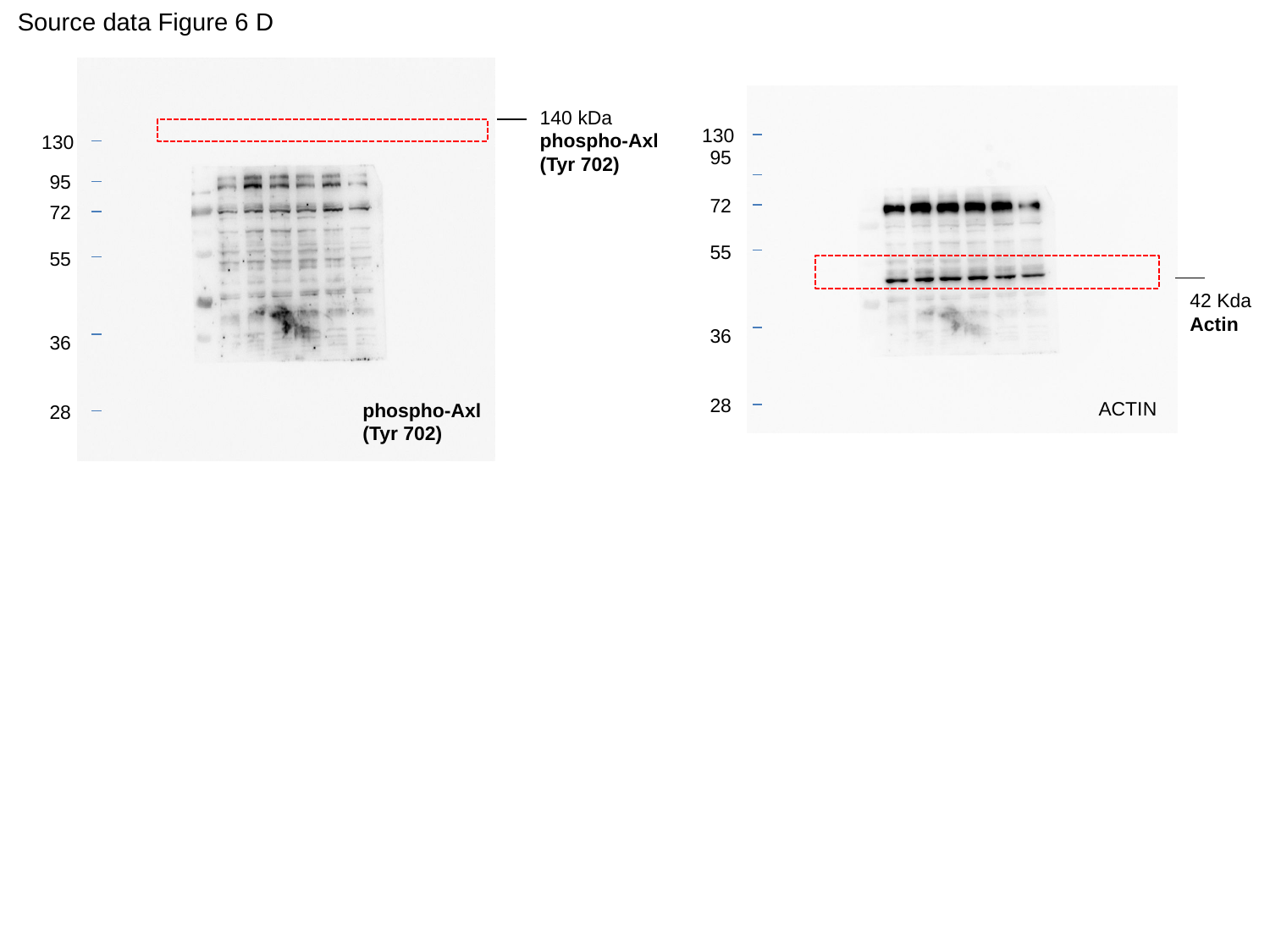

Source data Figure 6 D
140 kDa
phospho-Axl
(Tyr 702)
130
130
95
95
72
72
55
55
42 Kda
Actin
36
36
phospho-Axl
(Tyr 702)
28
 ACTIN
28

## Slide 3
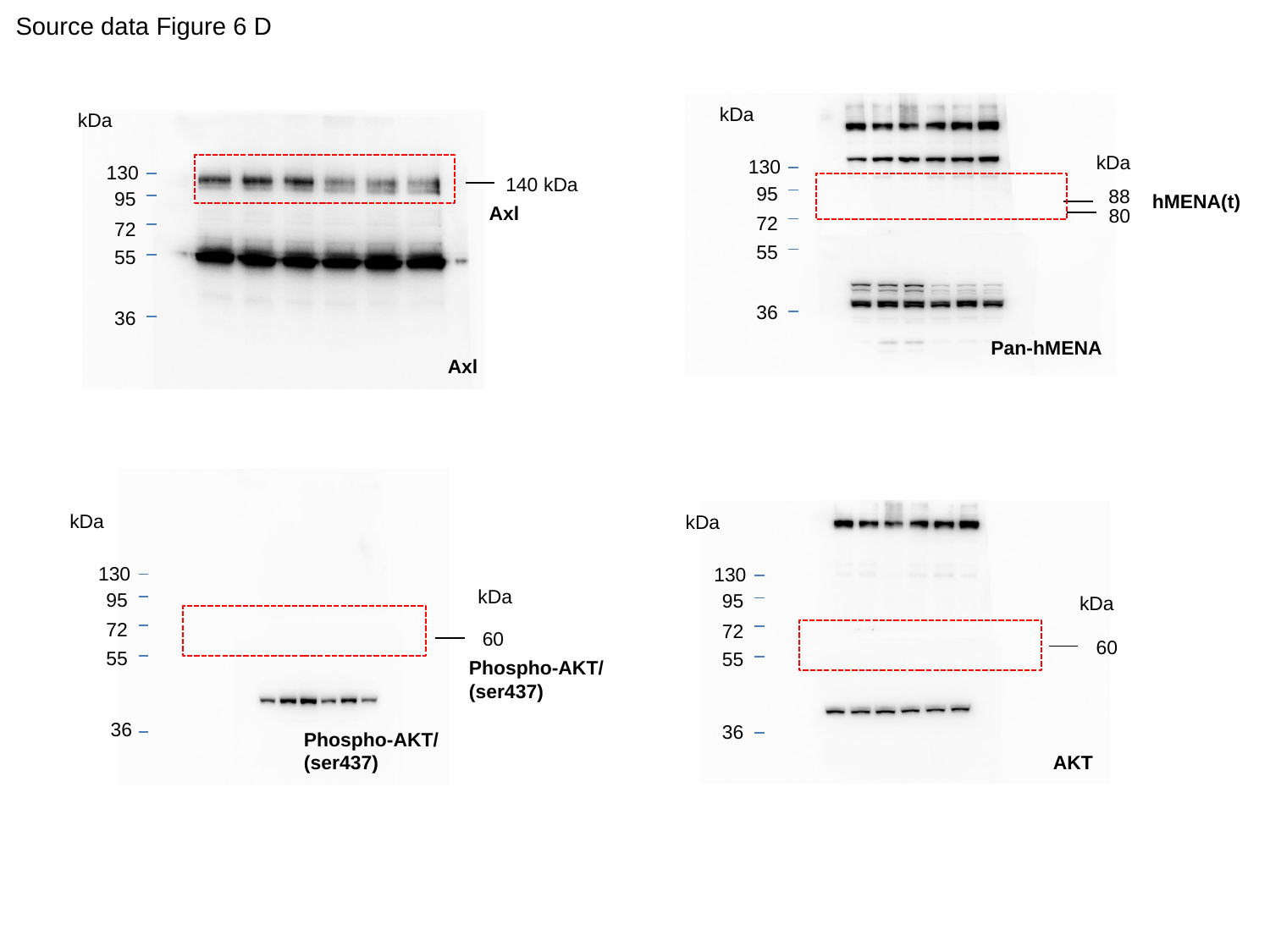

Source data Figure 6 D
kDa
130
95
72
55
36
kDa
130
95
72
55
36
kDa
140 kDa
88
hMENA(t)
Axl
80
Pan-hMENA
Axl
kDa
130
95
72
55
36
kDa
60
AKT
kDa
130
95
72
55
36
kDa
60
Phospho-AKT/
(ser437)
Phospho-AKT/
(ser437)

## Slide 4
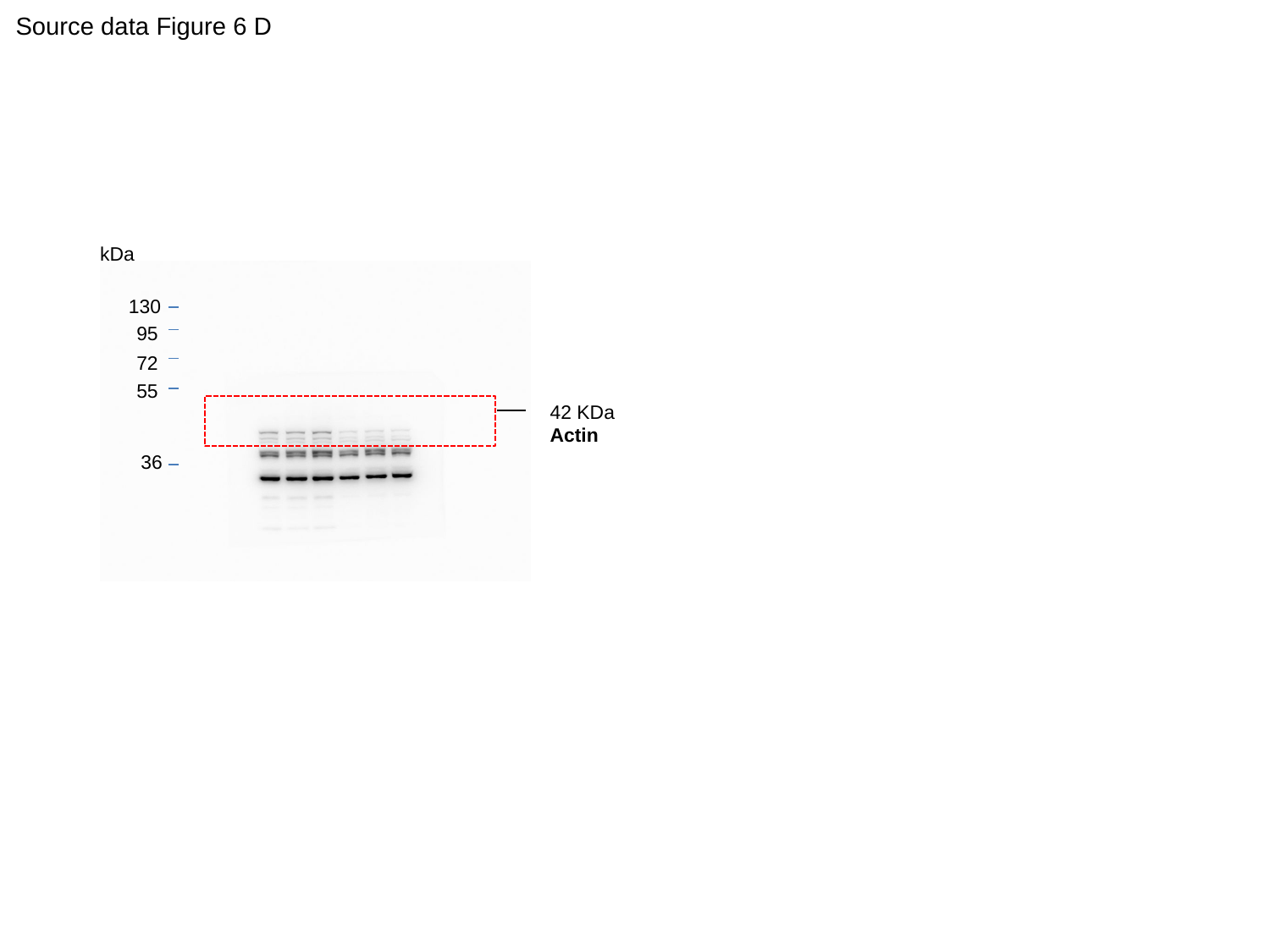

Source data Figure 6 D
kDa
130
95
72
55
36
42 KDa
Actin
